# One-step genome engineering in bee gut bacterial symbionts

**DOI:** 10.1128/mbio.01392-24

**Published:** 2024-08-06

**Authors:** Patrick J. Lariviere, A. H. M. Zuberi Ashraf, Lucio Navarro-Escalante, Sean P. Leonard, Laurel G. Miller, Nancy A. Moran, Jeffrey E. Barrick

**Affiliations:** 1 Department of Molecular Biosciences, The University of Texas at Austin, Austin, Texas, USA; 2 Department of Integrative Biology, The University of Texas at Austin, Austin, Texas, USA; Max Planck Institute for Chemical Ecology, Jena, Germany

**Keywords:** honey bee, *Snodgrassella alvi*, *Bartonella apis*, genome engineering, homologous recombination, microbiome

## Abstract

Mechanistic understanding of interactions in many host-microbe systems, including the honey bee microbiome, is limited by a lack of easy-to-use genome engineering approaches. To this end, we demonstrate a one-step genome engineering approach for making gene deletions and insertions in the chromosomes of honey bee gut bacterial symbionts. Electroporation of linear or non-replicating plasmid DNA containing an antibiotic resistance cassette flanked by regions with homology to a symbiont genome reliably results in chromosomal integration. This lightweight approach does not require expressing any exogenous recombination machinery. The high concentrations of large DNAs with long homology regions needed to make the process efficient can be readily produced using modern DNA synthesis and assembly methods. We use this approach to knock out genes, including genes involved in biofilm formation, and insert fluorescent protein genes into the chromosome of the betaproteobacterial bee gut symbiont *Snodgrassella alvi*. We are also able to engineer the genomes of multiple strains of *S. alvi* and another species, *Snodgrassella communis*, which is found in the bumble bee gut microbiome. Finally, we use the same method to engineer the chromosome of another bee symbiont, *Bartonella apis*, which is an alphaproteobacterium. As expected, gene knockout in *S. alvi* using this approach is *recA*-dependent, suggesting that this straightforward procedure can be applied to other microbes that lack convenient genome engineering methods.

**IMPORTANCE:**

Honey bees are ecologically and economically important crop pollinators with bacterial gut symbionts that influence their health. Microbiome-based strategies for studying or improving bee health have utilized wild-type or plasmid-engineered bacteria. We demonstrate that a straightforward, single-step method can be used to insert cassettes and replace genes in the chromosomes of multiple bee gut bacteria. This method can be used for investigating the mechanisms of host-microbe interactions in the bee gut community and stably engineering symbionts that benefit pollinator health.

## INTRODUCTION

Bacterial symbionts impact host health, including through their metabolic activities and by affecting how they respond to pathogens (1–3). In many systems, -omics-level studies, such as whole-genome sequencing (WGS), RNA sequencing (RNA-seq), or transposon sequencing (Tn-seq), have provided a high-level understanding of how specific symbionts interact with their hosts (4–9). However, achieving a deeper understanding of symbiosis requires tools for editing a symbiont genome to enable reverse genetics. While the chromosomes of certain animal symbionts (10–13) have been successfully engineered, a lack of easy-to-use approaches has hindered more extensive use of reverse genetics in many host-symbiont systems, including the honey bee microbiome.

Honey bees are critical pollinators in agriculture and essential for global food security (14–16). Moreover, it has become increasingly clear that the health of bees is influenced by their microbiome (3, 17–21). *Snodgrassella alvi* (*Neisseriaceae*) is one of about eight bacterial species that form the core gut microbiome present in all worker bees (3, 22). *S. alvi* colonizes the wall of the ileum (3, 23) and is postulated to deplete oxygen in the ileum and thereby create an anoxic environment for other community members (18). Experimental studies with gnotobiotic bees show that *S. alvi* protects against pathogens that infect the bee gut (24–26). *S. alvi* strains engineered with plasmids have been used to induce RNAi to perform functional genomics in bees (27, 28) and to combat bee pathogens such as *Nosema* and deformed wing virus (26, 27, 29). A handful of non-core bacteria, including *Bartonella apis* (1), are found in the gut communities of some but not all worker bees. Fundamental questions remain concerning how both core and non-core microbiome members, such as *S. alvi* and *B. apis*, colonize and interact with the bee host.

Many bacterial genome engineering approaches leverage homologous recombination (HR), using matching sequences in a DNA fragment to target an edit to the chromosome (30–34). Recombineering uses phage recombination machinery (35, 36) to facilitate efficient recombination in *Escherichia coli* and its close relatives and has the advantage of requiring only short homology segments (<50 base pairs). However, such reliance on the expression of exogenous machinery usually necessitates species-specific optimization to express an active recombinase when applying these methods to bacteria not closely related to *E. coli*, and this is not always possible. Another approach, suicide vector-assisted allelic exchange, has broader applicability than recombineering across bacterial clades (37–40). It does not require expression of exogenous recombinases, but requires long homology segments (>500 base pairs) in the donor DNA and can yield off-target edits. This procedure can be coupled to Cas9-directed counterselection, as was previously used to engineer *S. alvi* (41) to improve selection against single-crossover integration events, but this and similar multi-step processes are technically challenging, laborious, and have limited efficiency. A one-step approach to recombination that transforms cells with only linear DNA containing long homology segments has been described in Gram-positive bacteria (42–44) and Gammaproteobacteria (45, 46), particularly those exhibiting natural competence (44, 47, 48), but it is less common in other Pseudomonadota (formerly Proteobacteria).

Here, we engineer bacterial symbiont genomes in a manner that is one-step, independent of exogenous recombinase expression (lightweight), and not reliant on natural competence. We reliably and efficiently insert antibiotic cassettes, including with linked reporter genes, into the chromosomes of two distantly related bee gut symbiont groups, the betaproteobacterial genus *Snodgrassella* and the alphaproteobacterial species *B. apis*. In the immediate term, this approach will enable studies of symbiont-bee interactions and applications that engineer symbionts to protect bee health. Beyond the bee microbiome, this approach shows how recent advances in DNA synthesis and assembly can be used to implement a straightforward genome engineering workflow in diverse bacterial symbionts, ultimately, contributing to an improved understanding of host-microbiome interactions.

## RESULTS

### *S. alvi* genes can reliably be knocked out in one step

**Fig 1:**
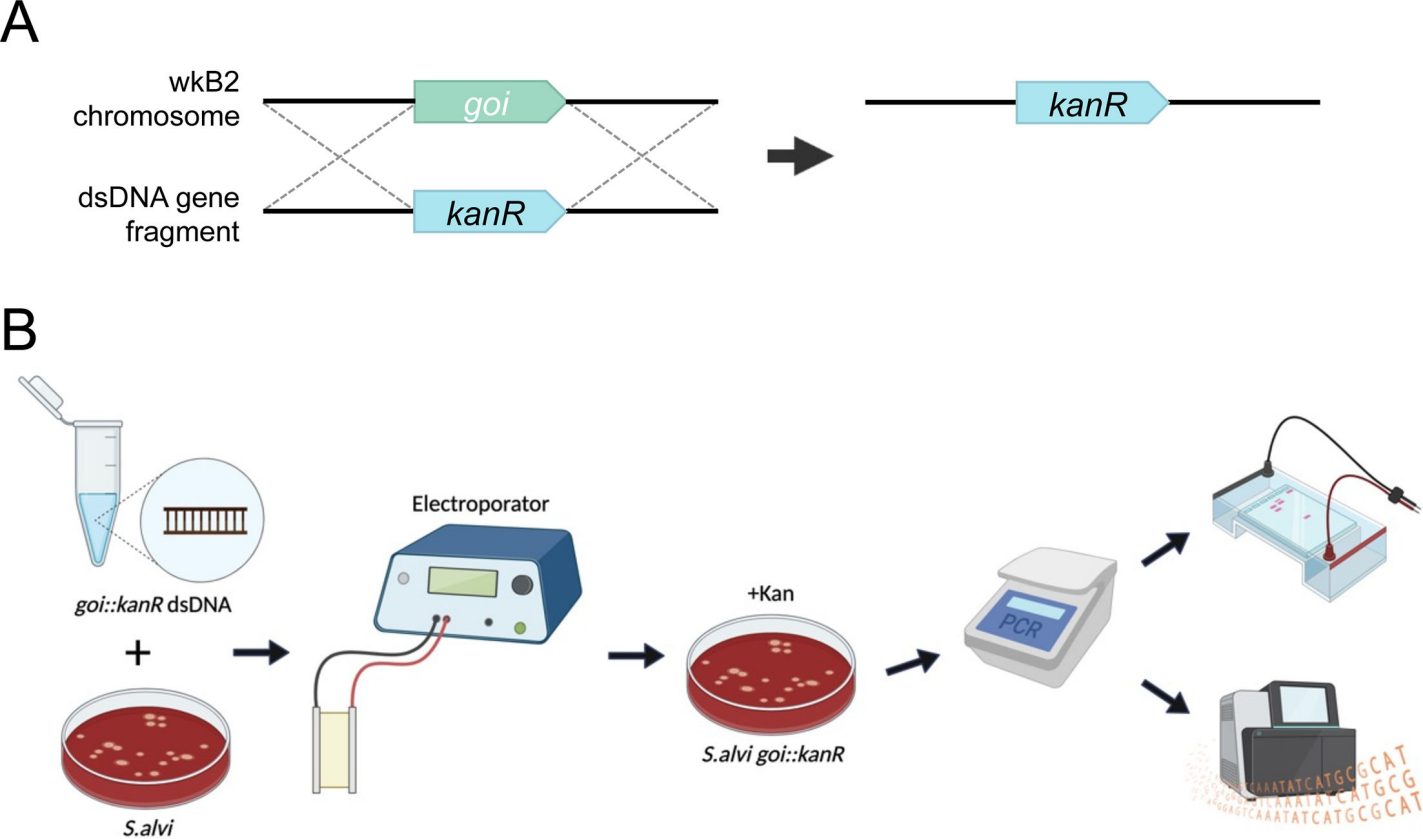
Genome engineering overview and workflow. (**A**) Cartoon depicting an overview of gene knockout by a lightweight genome engineering approach. An antibiotic resistance cassette, such as *kanR*, is designed to be flanked by arms with homology surrounding the deletion target. The targeted genomic locus is replaced by the antibiotic resistance cassette through single-step homologous recombination. (**B**) Diagram of genome engineering workflow. Five micrograms of linear or circular knockout construct DNA is electroporated into *S. alvi* wkB2. Following overnight liquid recovery with no antibiotics, transformants are selected on media containing antibiotics. Transformants are isolated and the deleted region is screened via PCR. Amplicons are purified, visualized on an agarose gel, and sequenced.

We sought to implement a targeted method for disrupting genes in *S. alvi* to study their functions and create engineered strains with new properties. Electroporation has been used for plasmid transformation in *S. alvi* wkB2 (49), so we asked if it could be used to deliver linear double-stranded DNA (dsDNA) into *S. alvi* cells to modify the chromosome through homologous recombination (Fig. 1A). In this workflow, a construct is designed with an antibiotic resistance gene flanked by long homology arms matching a gene of interest and/or adjacent sequences in the chromosome. Linear DNA constructs are chemically synthesized, assembled, and PCR amplified. Then, *S. alvi* wkB2 cells are electroporated with the dsDNA construct, recovered overnight, selected on antibiotic-containing media, and screened for proper insertion of the construct (Fig. 1B).

**Fig 2:**
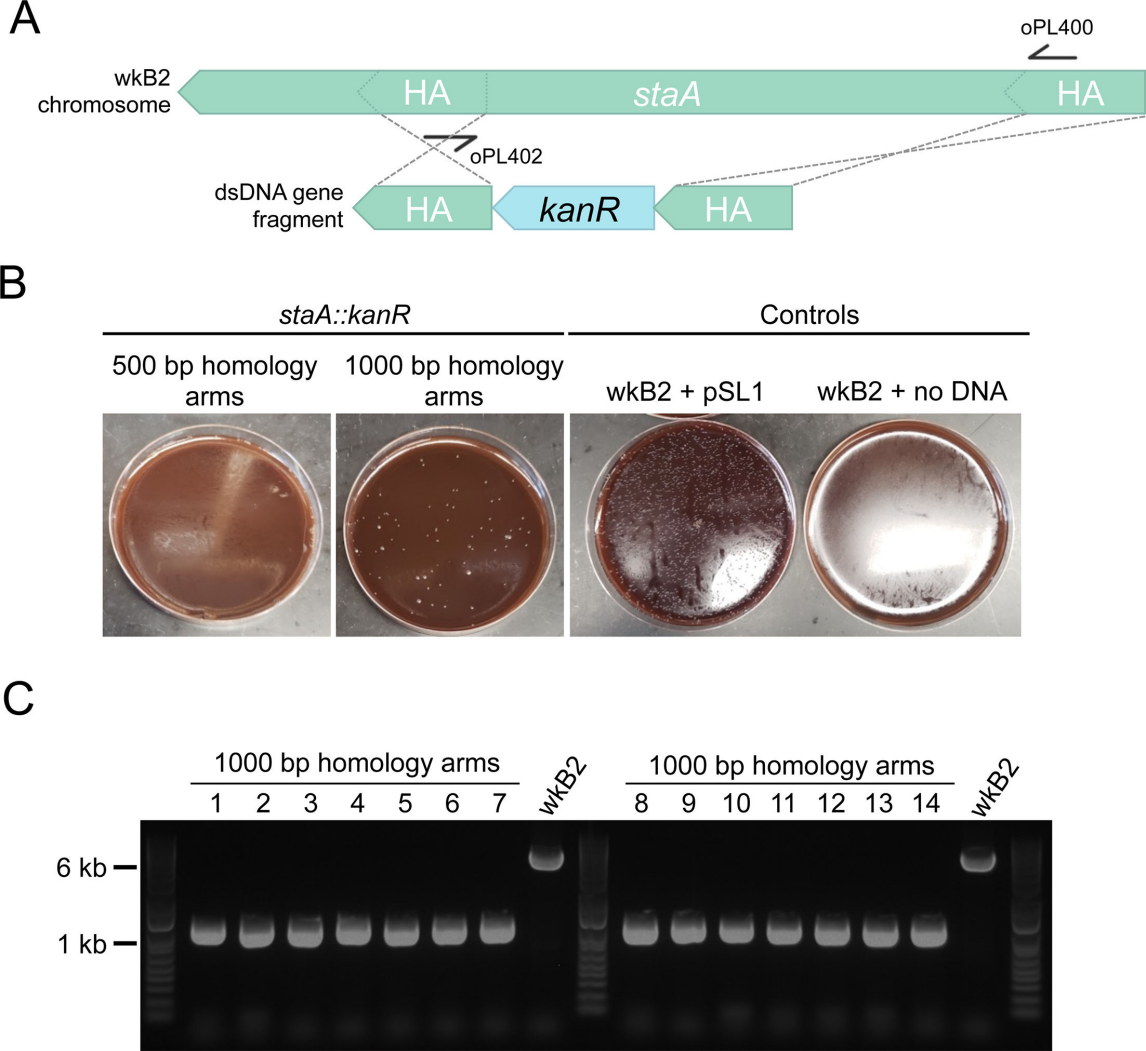
Gene knockout in *staA* is accurate. (**A**) Gene deletion map depicting genomic *staA* and the corresponding deletion construct. The deletion construct is composed of a *kanR* cassette flanked by 500 or 1,000 bp arms with homology internal to the *staA* gene. Primers oPL400 and oPL402 were designed for use in PCR screening for proper deletion. (**B**) Images of *staA::kanR* transformants and controls on Columbia + 5% sheep’s blood (Col-B) + kanamycin agar plates. wkB2 can be successfully transformed with a *staA::kanR* cassette containing 1,000 bp (middle left), but not 500 bp (left), homology arms, indicating that 1,000 bp of homology is needed to isolate colonies with the deletion. wkB2 cannot grow on kanamycin plates (right) without the presence of a kanamycin resistance cassette, as illustrated by control cells that received the *kanR*-containing pSL1 plasmid (middle right). (**C**) DNA gel of PCR screen for *staA* deletion. Large and small colonies of wkB2 + *staA::kanR* with 1,000 bp homology arms or wkB2 were amplified via PCR using oPL400 and oPL402, and run on a 1% agarose gel. The ~6,000 bp band observed in the wkB2 sample corresponds to the genomic region within *staA*, whereas the ~1,000 bp band observed in experimental samples corresponds to the *kanR* cassette. 100% of screened colonies were found to contain a single 1,000 bp band, indicating proper deletion of *staA*.

To pilot this approach, we targeted *staA* for deletion. This gene is non-essential (4) and was previously disrupted using the Cas9/suicide vector approach (41). We designed *kanR* knockout constructs with homology arms inside of the *staA* gene that were either 500 or 1,000 bp long (Fig. 2A). Initial electroporations using approximately 150 ng and 300 ng of DNA did not yield any transformants. Increasing the concentration of DNA to 5 µg proved sufficient for isolating kanamycin-resistant transformants (Fig. 2B). We found that 1,000 bp of homology yielded 85 transformants, whereas 500 bp of homology yielded none (Fig. 2B; Table S1). From the plate transformed with 1,000 bp homology arms, we picked 14 colonies. Colonies were screened via PCR for insertion of the *kanR* cassette using primers specific to sequences within the homology arms. All 14 of the screened colonies contained the *kanR* cassette inserted at the proper locus (Fig. 2C; Table S1), indicating on-target editing accuracy of 100% in the screened colonies. Short-read WGS of one of these *staA::kanR* strains confirmed that the targeted stretch of *staA* had been deleted and replaced with the *kanR* gene (Table S2). Finally, we confirmed efficient recombination of the 1,000 bp *staA::kanR* construct through a repeat electroporation. From these data, we calculated recombination efficiency to be 19.2 transformant CFU/μg DNA and determined the recombination frequency to be 4.34 × 10^−8^ transformant CFU/total cells (Table 1).

**TABLE 1:** Recombination efficiency and frequency

Genome modification	Recombination efficiency (transformant CFU/μg DNA)	Recombination frequency (transformant CFU/total cells)^*a*^
PEB0150 *ilvD::kanR*	3,989.6	n.d.
PEB0150 *ilvD::specR*	1,672.2	n.d.
PEB0150 *ilvD::ampR*	50.4	n.d.
PEB0150 *ilvD::gfp-ampR*	816.6	n.d.
wkB2 *staA*::*kanR*	19.2	4.34 × 10^−8^
wkB2 *pilF*::*kanR*	107.8	3.83 × 10^−7^
wkB2 *pilG*::*kanR*	79	3.93 × 10^−7^
wkB2 *recA*::*kanR*	294.2	1.37 × 10^−6^
wkB2 *recJ*::*kanR*	295.8	2.21 × 10^−6^
wkB2 *amsE*::*kanR*	349.8	2.17 × 10^−6^
wkB2 *asr*::*kanR*	216.3	2.91 × 10^−6^
wkB2 SAL_WKB2_RS11215/SAL_WKB2_RS11220 intergenic space::*gfp-kanR*	>20	n.d.
wkB2 SAL_WKB2_RS11215/SAL_WKB2_RS11220 intergenic space::*kanR-e2 crimson*	12	n.d.
wkB2 *clpA*::*kanR-e2 crimson*	10	n.d.
wkB9 *modA/fdnG::specR*	1.544 × 10^4^	n.d.
wkB332 *modA/fdnG::specR*	127	n.d.

^*a*^ n.d., not determined.

To further assess the reliability of this approach, we designed and tested knockout constructs for six additional non-essential (4) *S.alvi* genes: *pilF*, *pilG*, *asr*, *asmE*, *recJ*, and *recA*. Similar to the *staA* knockout construct, we designed kanamycin resistance cassettes flanked by 1,000 bp homology arms (Fig. S1). This time, the homology arms were designed to match regions upstream and downstream of the CDS, with 10 bp of CDS left on 5′ and 3′ ends, so that nearly the whole gene would be deleted (Fig. S1). As before, dsDNA constructs were synthesized and electroporated into *S. alvi* wkB2. Then, cells were plated on antibiotic-containing media to select for integration of the cassette into the chromosome. The number of transformants in this initial trial ranged from ~20 to >100 colonies, depending on the gene targeted for knockout (Table S1). Because PCR screening showed that editing accuracy was 100% for the *staA* knockout, we screened fewer clones for successful deletion for these six additional knockouts. For five of the six knockouts, 100% of clones had successful recombination, as determined by PCR or WGS (Fig. S2; Table S1). For the remaining gene, *asr*, a PCR screen confirmed recombination in two of the four clones (Fig. S2; Table S1). PCR of the other two clones yielded no band the same size as the insertion or native gene, possibly indicating that the PCR, rather than recombination, failed. WGS confirmed each of these six genes was knocked out and successfully replaced by the kanamycin resistance cassette (Table S2). Finally, we repeated electroporation of each of these constructs and calculated recombination efficiency. Recombination efficiency ranged from 79 to 350 CFU/μg DNA, and recombination frequency was as high as 2.91 × 10^−6^ transformant CFU/total cells (Table 1). In aggregate, these data demonstrate the feasibility of scaling up this genome engineering workflow to construct multiple *S. alvi* knockout strains in parallel, with near-perfect on-target editing reliability.

A handful of secondary mutations, including point mutations, base substitutions, or short deletions located far away from the site of antibiotic resistance cassette insertion, were observed in engineered strains (Tables S2 and S3). We suspect that many of these mutations were not directly caused by addition of insertion cassettes, as we were previously aware of some pre-existing mutations in our *S. alvi* stock that evolved during laboratory propagation after deposition of the genome sequence. Regardless, we still wanted to assess any effect these mutations might have on interactions with the host. To test if four of the commonly mutated genes (Table S2, PL238 tab) had an impact on establishment in hosts, we colonized newly emerged adult bees with wkB2 or *ampD::kanR* cells, which had secondary mutations in the above four genes. After incubation of colonized bees for 7 days, CFU counts of dissected ilea on tetracycline-containing media showed that Δ*ampD* cells colonized bees to the same titer as wkB2 (wkB2 and wkB2-derived cells are tet-resistant) (Fig. S3). Thus, these four secondary mutations do not appear to impair host colonization.

### *S. alvi* type IV pilus mutants have impaired biofilm formation

**Fig 3:**
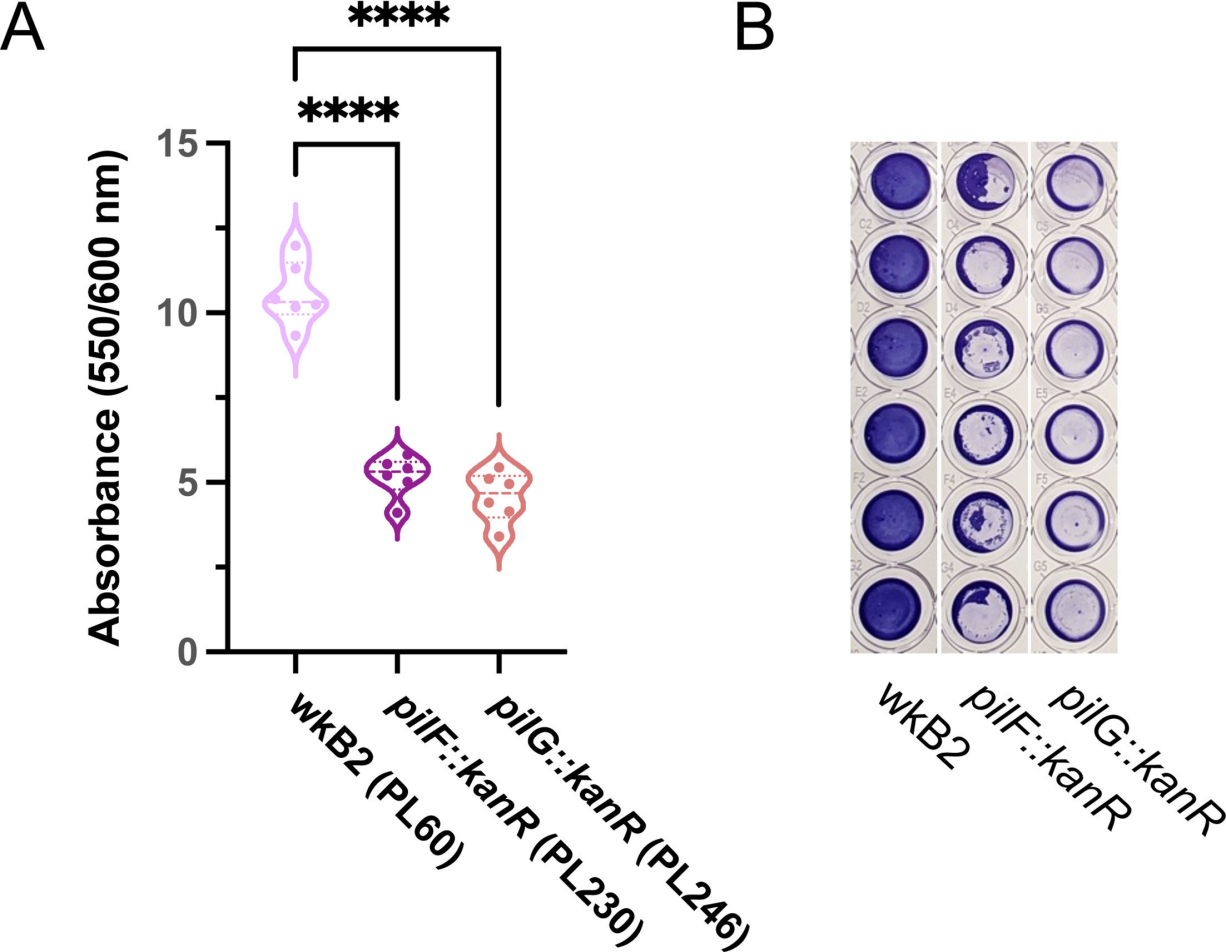
Phenotypic validation: Knockout of genes encoding adhesion proteins results in biofilm formation defects. (**A**) Plot of OD_550_/OD_600_ to quantify biofilm formation normalized to cell growth for biofilm knockout strains. Compared to WT, *pilF::kanR* and *pilG::kanR* have decreased biofilm formation relative to growth. Thick dotted line = median; thin dotted line = quartiles. *N* = 6 for each condition. ****: *P* < 0.0001, as determined by one-way parametric ANOVA with Dunnett’s multiple comparison test. (**B**) Image of crystal violet-stained 96-well plate columns demonstrating *pilF::kanR* and *pilG::kanR* mutants have reduced biofilm formation as compared to wkB2.

Biofilm formation has previously been used as a convenient phenotype for validating genome engineering tools (50). Two of the *S. alvi* knockout strains we created had deletions of type IV pilus genes, *pilF* and *pilG*. Biofilm formation was quantified for wild-type wkB2 and the *pilF::kanR* and *pilG::kanR* mutants using a crystal violet assay, by assessing OD_550_/OD_600_ of washed cells grown in 96-well plates. We found that knockout of both *pilF* and *pilG* led to a significant and visually noticeable decrease in biofilm formation compared to the wild-type strain (Fig. 3; Fig. S4), similar to that observed for mutant strains containing *pilF* and *pilG* genes disrupted by untargeted transposon insertions in a prior study (4). This result indicates our approach can be used to generate knockouts for downstream phenotype testing.

### Heterologous genomic insertions can be made using circular plasmid DNA

We next tested inserting heterologous genes into the genome and genome modification using circular DNA, as opposed to linear DNA. We approached these problems simultaneously by assembling constructs for inserting fluorescent protein-coding genes into the *S. alvi* chromosome on *E. coli* plasmids that do not replicate in *S. alvi*. Recombination using plasmid DNA would allow for knockout cassettes to be constructed from part plasmids using the bee microbiome toolkit (BTK) assembly scheme (41), which would enable re-use of the same homology flank parts and integrating different payloads into the chromosome without synthesizing DNA from scratch each time.

**Fig 4:**
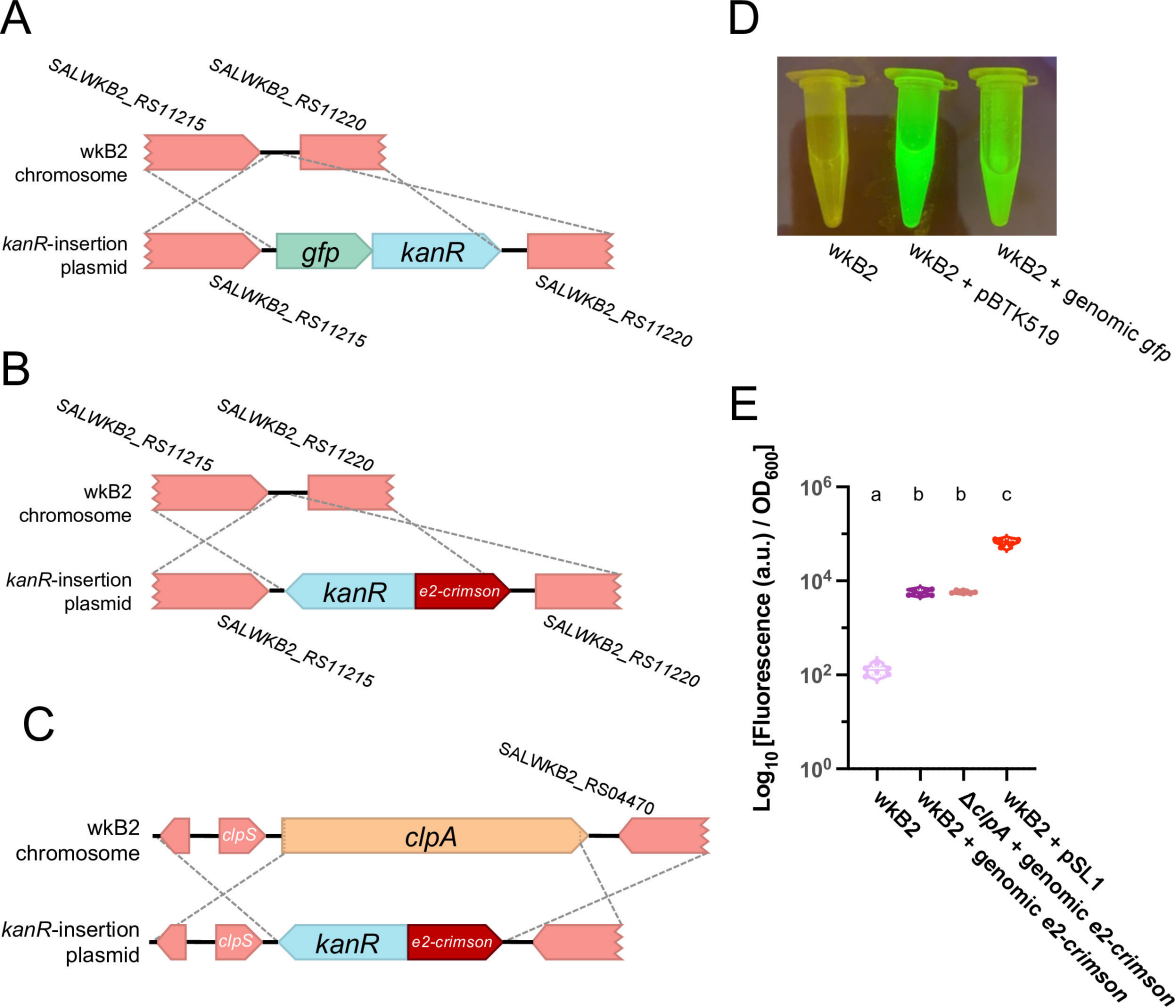
Fluorescent protein-coding genes can be inserted into the *S. alvi* genome. (**A**) Gene insertion map depicting insertion of a *gfp + kanR* cassette into a non-essential, intergenic region of the genome. (**B**) Gene insertion map depicting insertion of an *e2-crimson + kanR* cassette into a non-essential, intergenic region of the genome. (**C**) Gene insertion map depicting deletion of the *clpA* gene via replacement insertion of an *e2-crimson + kanR* cassette. (**D**) Image of wkB2, wkB2 + pBTK519 (positive control), and wkB2 + genomic *gfp* cells in front of a blue light transilluminator (470 nm), demonstrating successful insertion of *gfp* as evidenced by green fluorescence that is not observed in WT cells. (**E**) Plot of E2-Crimson fluorescence, demonstrating that wkB2 + genomic *e2-crimson* cell pellets resuspended in PBS display significantly increased red fluorescence compared to wkB2 and approximately one-tenth the fluorescence of wkB2 + pSL1, which contains a multicopy plasmid expressing *e2-crimson*. Log_10_ of OD_611 ex, 646 em_/OD_600_ is shown for cell cultures that were pelleted, resuspended in PBS, and read in a 96-well plate. Solid line = median; thin dotted line = quartiles. *N* = 6 for each condition. Dissimilar letters above each group indicate a significant difference in means (*P* < 0.0001, as determined by one-way parametric ANOVA with Tukey’s multiple comparison test).

We first identified an intergenic region of the wkB2 genome, situated between the non-essential genes *SALWKB2_RS11215* and *SALWKB2_RS11220* (4) as a likely neutral location to use as a landing pad. Then, we engineered part plasmids containing genes encoding one of two fluorescent proteins (GFP or E2-Crimson) and an antibiotic resistance gene, as well as homology arm part plasmids (Fig. 4A and B; Fig. S5 and S6). Parts were assembled into a single plasmid via Golden Gate assembly and cloned into *E. coli* (41). The purified, non-linearized plasmid was then electroporated into *S. alvi*, followed by isolation of antibiotic-resistant colonies (Fig. S5 and S6). We calculated recombination efficiencies ranging from 12 to >20 transformant CFU/μg DNA (Table 1; Table S1). Insertion of just the cassette into the proper genomic location without any other plasmid sequences remaining was confirmed by WGS (Table S2).

Next, we validated the phenotypes of the *gfp* and *e2-crimson* chromosomal integration strains. *S. alvi* wkB2 and wkB2 + *gfp* cells were grown in liquid culture and imaged on a blue light transilluminator. wkB2 + *gfp* cells were fluorescent, in contrast to wkB2 control cells (Fig. 4D), indicating that this chromosomally encoded *gfp* is capable of being successfully transcribed and translated into protein with observable fluorescence. To test the expression of *e2-crimson*, liquid cultures and phosphate-buffered saline (PBS)-resuspended pellets of wkB2, wkB2 + *e2-crimson*, and wkB2 + pSL1-E2C (51) (which contains plasmid-encoded *e2-crimson*) were quantified for E2-Crimson fluorescence normalized to cell growth (OD_600_). wkB2 + genomic *e2-crimson* had significantly higher fluorescence than wkB2 (Fig. 4E; Fig. S7), indicating successful production of chromosomally encoded *e2-crimson*. As expected for one genomic copy versus a multicopy plasmid, wkB2 + genomic *e2-crimson* produced less fluorescence than did wkB2 + pSL1 (Fig. 4E). These results indicate that exogenous genes can be successfully incorporated into the *S. alvi* genome.

Next, we demonstrated that a gene knockout could be made at the same time as a functional genomic insertion. To this end, we combined insertion of a fluorescent protein-coding gene with a deletion targeting the non-essential *clpA* gene. We designed a construct containing *e2-crimson* and *kanR*, flanked by arms with homology upstream and downstream of *clpA* (Fig. 4C; Fig. S8). We electroporated this construct and obtained transformants at an efficiency of 10 transformant CFU/μg DNA (Table 1; Table S1). Proper genomic insertion was confirmed by WGS (Table S2). Similar to cells containing a fluorescent protein-coding gene inserted into an intergenic region, we found that Δ*clpA + e2-crimson* cells were fluorescent, with no significant difference between these strains (Fig. 4E). This result confirms that fluorescent protein-coding gene insertion can be combined with gene deletion.

### DNA integration is RecA-dependent

**Fig 5:**
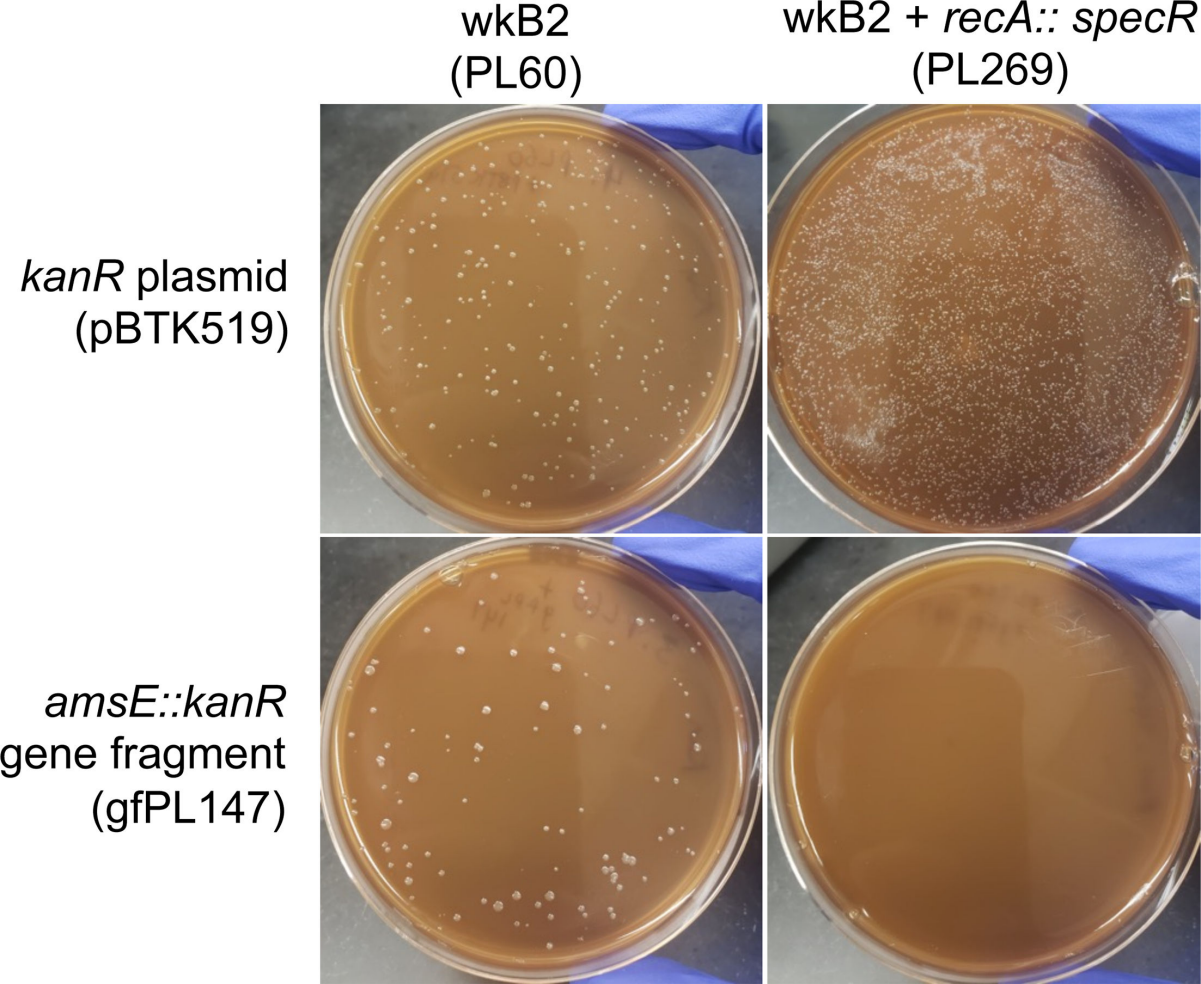
Gene knockout is *recA-*dependent. Plates demonstrating that wkB2 (PL60; top left) and wkB2 + *recA::specR* (PL269; top right) are successfully electroporated with a control *kanR* plasmid (pBTK519). The knockout construct *amsE::kanR* (gfPL147) is incorporated into wkB2 (bottom left), but not wkB2 + *recA::specR* (bottom right).

Having validated our genome engineering approach, we wanted to verify that genome integration was occurring through the expected RecA-mediated recombination mechanism that predominates in bacteria. In other species, dependence on RecA-mediated homologous recombination is routinely tested by attempting to recombine exogenous DNA in a *recA* knockout (52–61). Accordingly, we generated a *recA::specR S. alvi* strain and then attempted to delete another gene, *amsE*, in this background using a *kanR* cassette. We found that, while *recA::specR* could still be transformed with a plasmid and *amsE* could be knocked out in WT wkB2, *amsE* could not similarly be knocked out in the *recA::specR* strain (Fig. 5). To ensure that synthetic lethality between *asmE* and *recA* was not what accounted for the lack of transformants, we repeated this experiment by trying to knock out *recJ* in a *recA::specR* background. As before, *recJ* could be knocked out in wild-type *S. alvi* wkB2, but not in *recA::specR* cells (Fig. S9). This finding indicates that the lack of double mutants in a *recA::specR* background is likely not due to synthetic lethality.

In some bacterial species, such as *E. coli*, Δ*recA* cells display substantially decreased fitness, which could affect the number of colonies observed after transformation (62). We therefore asked if *S. alvi* Δ*recA* cells might be recombination deficient due to reduced fitness. We found the growth rate of our engineered *recA::kanR* strain to actually be enhanced compared to WT wkB2 (Fig. S10A), although this growth enhancement may be due to the presence of secondary mutations (Table S2). Furthermore, cell viability was similar for *recA::kanR* and WT cells (Fig. S10B). These data demonstrate that the inability to knock out genes in the Δ*recA* background is not due to reduced fitness, but instead due to the loss of *recA* function.

### Other *Snodgrassella* strains and species can be engineered

To test the functionality of our genome engineering approach in other *S. alvi* strains, we attempted to engineer two additional *S. alvi* strains, wkB9 and wkB332, both of which are also honey bee symbionts (63). An intergenic region between the non-essential genes *modA (SALWKB2_RS03155*) and *fdnG (SALWKB2_RS03160*) (4) was chosen for antibiotic resistance gene insertion (Fig. S11). Subsequently, we electroporated wkB9 and wkB332 with a plasmid containing an antibiotic resistance cassette flanked by the appropriate homology arms. Both transformations were successful (Fig. S12), with recombination efficiencies ranging from 127 to 1.544 × 10^4^ transformant CFU/μg DNA (Table 1; Table S1). Insertion of the cassette into the proper genomic location in each strain was confirmed by WGS (Table S2). These results indicate that our genome engineering workflow can be used in multiple strains of *S. alvi*.

Next, we tested engineering the genome of another species of *Snodgrassella*, *S. communis* wkB12, a bumble bee gut symbiont (63). We attempted to insert exogenous DNA into an intergenic region between the *SALWKB12_RS02015* and *SALWKB12_RS02020* genes (Fig. S13A). A plasmid containing an antibiotic resistance cassette and *gfp*, flanked by homology arms, was successfully electroporated into *S. communis*. Successful integration was initially assessed visually, as wkB12 + *gfp* cells displayed a significant increase in fluorescence under blue light compared to WT wkB12 cells (Fig. S13B and C). Then, insertion of the *gfp-specR* cassette in the expected location in the wkB12 genome was confirmed by WGS (Table S2). These results indicate that our approach is functional in another *Snodgrassella* species.

### The alphaproteobacterial bee gut symbiont *Bartonella apis* can be engineered

**Fig 6:**
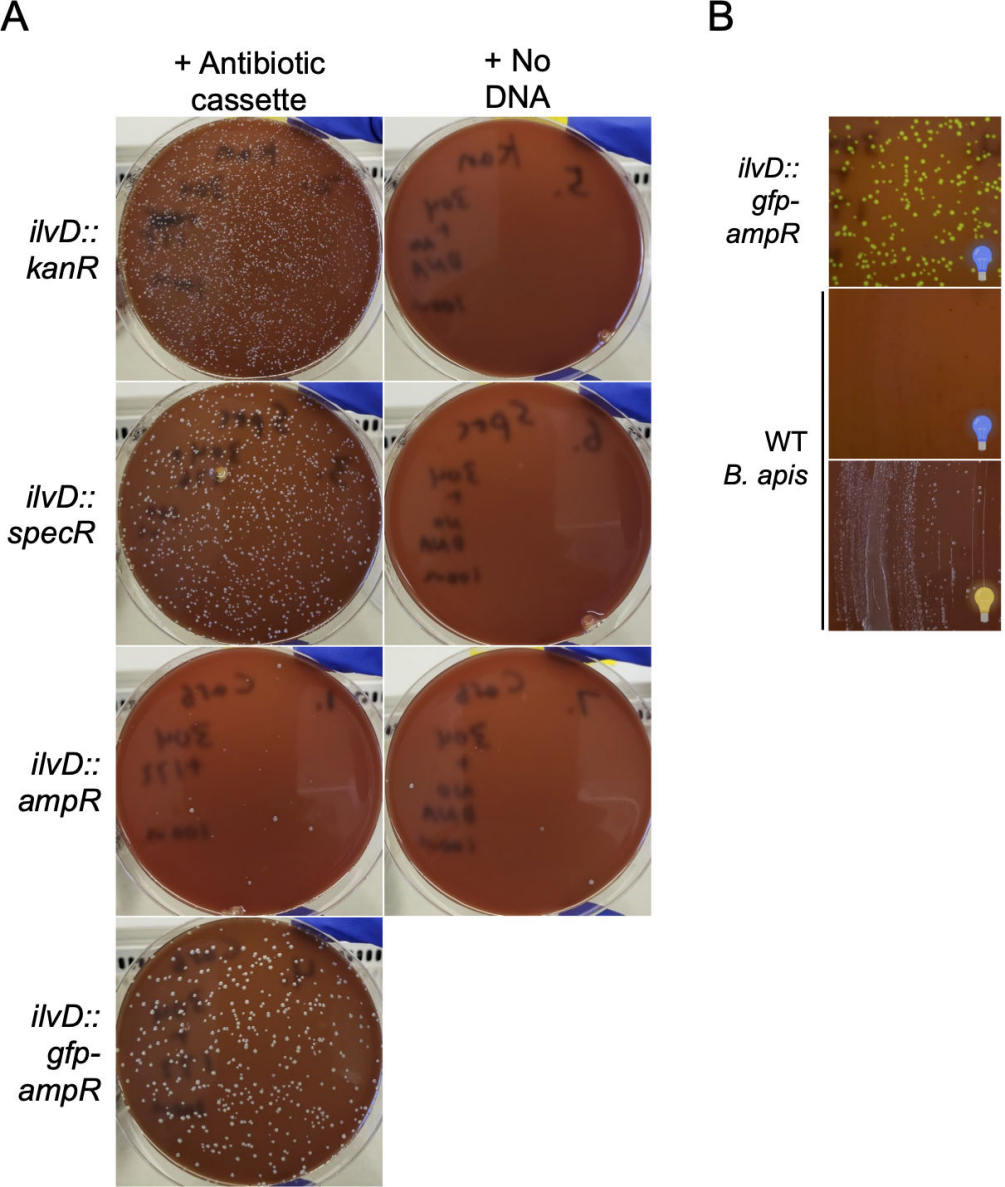
The *B. apis* genome can be engineered with a lightweight approach. (**A**) Plates demonstrating that *ilvD* can be knocked out in *B. apis*. Col-B + kan agar plates inhibit WT *B. apis* growth (top right) and select for insertion of *ilvD::kanR* (top left). Col-B + spec agar plates inhibit WT *B. apis* growth (middle-top right) and select for insertion of *ilvD::specR* (middle-top left). Col-B + carb agar plates inhibit most WT *B. apis* growth (middle-bottom right; a few spontaneously resistant mutants colonies are present) and select for insertion of *ilvD::ampR* (middle-bottom left) and *ilvD::gfp-ampR* (bottom left). (**B**) Plates illustrating successful *ilvD::gfp-ampR* transformation into *B. apis. ilvD::gfp-ampR* colonies fluoresce green in front of a blue light transilluminator (top), whereas WT *B. apis* (bottom) does not not fluoresce (middle). See panel A (bottom left) for *ilvD::gfp-ampR* colonies imaged under ambient light. Blue light bulb: plate imaged in front of blue light; yellow light bulb: plate imaged under ambient light.

To test the broader applicability of our approach, we first attempted plasmid electroporation in another symbiont. We successfully electroporated *Bartonella apis*, a non-core bee gut microbiome member (64, 65), with an *ampR* plasmid (Fig. S14). We then attempted to make gene deletion/insertions in *B. apis* PEB0150 (65) in the gene *ilvD. ilvD* was selected for knockout because it was found to be non-essential in a Tn-seq screen in another member of the genus, *Bartonella henselae* (66). We designed and amplified four constructs to replace *ilvD* with either *ampR*, *kanR*, *specR*, or *gfp-ampR*, to control for the possibility that different antibiotics would be better for engineering *B. apis* (Fig. S15). We then electroporated *B. apis* with one of the four constructs or no DNA and plated on selective media. We successfully isolated transformants for all four constructs (Fig. 6). A PCR screen for cassette insertion found one construct (*ilvD::ampR*) had 100% recombination accuracy and two other constructs (*ilvD::kanR* and *ilvD::specR*) had the expected integrations in seven of the eight screened colonies (Fig. S16). In these two transformations a single clone yielded no band, possibly indicating the PCR reaction failed. Recombination efficiency in the fourth construct (*ilvD::gfp-ampR*) was 99.5%, as determined by comparing fluorescent to non-fluorescent colonies (Fig. 6; Fig. S16). To confirm insertion of each construct at the proper genomic locus, we characterized engineered *B. apis* strains by WGS. We found that each construct successfully knocked out the intended gene (Table S2). As with the engineered *S. alvi* strains, we saw a handful of secondary mutations (not near the insertion cassette) in engineered *B. apis* strains (Table S2), presumably due to either suppression of *ilvD* deletion or adaptation to laboratory culture conditions. Overall, the recombination and sequencing results demonstrate that the genome of *B. apis*, a bee gut symbiont not closely related to *S. alvi*, can also be engineered. Taken together with the *S. alvi* engineering results, we conclude that our approach has potential for engineering the genomes of diverse symbionts.

## DISCUSSION

Here, we show that a one-step, lightweight approach for engineering bacterial symbiont chromosomes using electroporation and homologous recombination works in multiple bee gut symbionts. Previous strategies for genetically engineering insect symbionts have had varied success (10). While transposon mutagenesis has been described in a number of species (4, 10, 12, 67, 68), it is not site-specific. Site-specific deletions have been constructed in a handful of insect symbionts, through expression of exogenous lambda Red machinery (BfO2, *Sodalis glossinidius*) (69, 70) or use of a two-step suicide vector strategy (*R. rhodnii*, *S. glossinidius*, *Burkholderia insecticola*, *S. alvi*, *B. apis*, and *Frischella perrara*) (10, 12, 41, 69, 71, 72), both of which add either complexity or time to knockout construction. Compared to symbiont engineering described in other insect systems, the approach described here is straightforward, flexible, and reliable.

Transforming cells with DNA fragments that have long homology to the target chromosome in order to integrate an antibiotic resistance cassette is a well-established approach for bacterial genetics (36). Recently, it has become easier to create DNA constructs with the large homology regions necessary for efficient integration through native recombination pathways. One reason is the widespread availability and increasing affordability of commercial services for synthesizing and assembling multi-kilobase DNA fragments (73). Alternatively, and as we also show, standardized kits for assembling genetic parts into plasmids also make it possible to more easily assemble these large constructs. We anticipate this approach will be useful for engineering the genomes of additional bacteria, both symbionts and non-symbionts.

Bottlenecks and key questions remain as far as predicting when this method will be successful. For it to work, symbionts must be culturable and there must be a way to effectively deliver DNA into cells. Furthermore, it is unclear to what extent the success of this approach is influenced by species-specific propensities for recombination and genomic defenses against foreign DNA. *B. apis* recombination efficiency, for example, was up to an order of magnitude higher than that of *S. alvi* wkB2. Future work will address these questions, but success in engineering diverse genomes suggests the potential for applicability in diverse Pseudomondota.

Recombination efficiency in other bacteria (typically non-symbionts) can range widely, depending on the species and use of exogenous machinery such as lambda Red proteins. For example, recombination efficiencies of 10^1^ CFU/µg (*A. baumannii*) (50), 10^0^–10^3^ CFU/µg (lactic acid bacteria) (74), 10^4^ CFU/µg (*B. subtilis*) (75), and 10^5^ CFU/µg (*E. coli*) (76) have been reported. Accordingly, *S. alvi*’s recombination efficiencies of 10–350 CFU/μg and *B. apis*’s recombination efficiency of 50–4, 000 CFU/µg (Table 1) are within the range of reported values for other species. Recombination frequencies in other species can range from at least 10^−7^ to 10^−4^ (53, 56, 77). Aside from the *staA::kanR* construct, which transformed into wkB2 at the lower frequency of 10^−8^ (Table 1), all of the other constructs tested for *S. alvi* wkB2 fell within the 10^−7^ to 10^−6^ recombination frequency range (Table 1). The recombination efficiency and frequency values we observe, therefore, are in line with those observed in other bacterial species.

Mechanistically, integration of a circular plasmid into a genome is typically a two-step process (78), with each crossover occurring sequentially. Previous modification of the *S. alvi* genome using conjugation of an R6K suicide vector resulted in mostly single-crossover integrations of the plasmid, and targeting Cas9 cleavage to the integration site was needed to increase the fraction of cells with double-crossover integration (i.e., just integration of the antibiotic resistance gene versus the entire plasmid) (41). However, we observed only double-crossover integrants when we electroporated a ColE1 plasmid that cannot replicate in *S. alvi*, even when we did not linearize the plasmid. This discrepancy may be due to how conjugation delivers single-stranded DNA to a cell versus the dsDNA that was electroporated. Apparently, *S. alvi* recombination is efficient enough to favor the double-crossover integration, which makes linearization of the plasmid prior to electroporation unnecessary.

For each of the *S. alvi* and *B. apis* knockout strains we made, we observed secondary mutations in the whole-genome sequencing results (Table S2 and S3). Some of these mutations are present in our ancestral stock or are commonly mutated, whereas others are found in only one engineered strain. Because these secondary mutations arise due to short deletions, insertions, or point mutations distal from the intended cassette insertion site, they appear to be passenger mutations likely not specifically caused by our genome engineering approach. No engineered strains were found to have antibiotic resistance cassettes inserted off-target, leading us to conclude this approach has high engineering accuracy. We suspect that non-ancestral secondary mutations could arise as suppressors to genes targeted for deletion or due to adaptation to lab culture conditions, such as growth in liquid media. Historically, whole-genomes have rarely been sequenced after performing genome engineering procedures, so it is unclear whether this represents normal variation or something mutagenic about the procedure. Regardless, these secondary mutations did not appear to affect symbiosis between *S. alvi* and the bee (Fig. S3).

In their current form, our results provide immediate value to the bee microbiome field. This approach will facilitate study of the roles of *Snodgrassella* and *B. apis* in host-symbiont interactions by reverse genetics, allowing mechanistic investigations of host engraftment, immune stimulation, and nutrient metabolism. Our approach will also enable investigations of how symbionts interact with other microbiome members and bee pathogens. Further, we foresee chromosomal integration making new bee gut symbiont engineering projects possible, ranging from migrating plasmid-based machinery to the chromosome to constructing gain-of-function mutant strains that improve upon *S. alvi*’s probiotic capabilities (79, 80). Finally, the interoperability of our genome engineering approach and the BTK (41) can expedite strain construction. Part plasmids constructed to contain homology arms can be mixed and matched with different payloads, allowing for streamlined knockout/insertion library construction.

With further development, new use cases and features could expand the utility of our approach. Induction/depletion experiments (performed either with a gene knockout and complementation with a plasmid-based copy of a gene under control of an inducible promoter, or by placing an inducible promoter into the genome upstream of a gene) could be performed *in vivo* (49) to study essential cellular processes on demand. Use of site-specific recombinases such as the Flp-FRT system (81) could remove confounders (such as the presence of antibiotic resistance cassettes) in attributing phenotypes to gene deletions and allow recycling antibiotic resistance cassettes to make multiple edits. Expression of exogenous recombineering systems (e.g., RecET or lambda Red) from a plasmid could improve recombination efficiency, as in *E. coli* or *Acinetobacter baumanii*, for example (35, 50, 82). Finally, allelic exchange, facilitated by two-step recombination and use of counter-selectable markers (83), would allow for study of loss-of-function or gain-of-function mutants. In short, we expect the approach presented in this paper to not only have immediate utility in reverse genetics and engineering applications in bee gut symbionts, but to also inform efforts and possibly serve as a template for engineering the genomes of other bacteria.

## MATERIALS AND METHODS

### Strains and DNA constructs

Strains, plasmids, oligos, and gene fragments are reported in Table S4.

### Bacterial cell culture

*S. alvi*, *S. communis*, and *B. apis* cells were grown on Columbia + 5% sheep’s blood (Col-B) agar plates (with or without antibiotics, as indicated) at 35°C, 5% CO_2_. Cultures were typically incubated for 2–3 days (*S. alvi*), 3–4 days (*B. apis*), or 4–5 days (*S. communis*). *S. alvi* cells were grown in liquid culture (with or without antibiotics, as indicated) at 35°C, 5% CO_2_, with 2–3 day incubation time. Media and containers used for liquid culture depended on the application, as indicated below. *E. coli* cells were grown with antibiotics on LB + 1.5% agar plates or in liquid LB, at 37°C, incubating overnight. Where indicated, 25 µg/mL of kanamycin (kan) was used in *S. alvi* cultures and 20 µg/mL was used in *B. apis* cultures; 30 µg/mL of spectinomycin (spec) was used in *S. alvi*, *S. communis*, and *B. apis* cultures; 60 µg/mL of spectinomycin was used in *E. coli* cultures. 30 µg/mL of carbenicillin (carb) was used in *B. apis* and *E. coli* cultures. Media containing 0.3 mM 2,6-diaminopimelic acid (DAP) was used to grow DAP-auxotrophic *E. coli* (PL280). Strains were saved in 30% glycerol stocks and stored at −80°C. Plasmids were obtained from *E. coli* overnight cultures using a Monarch plasmid miniprep kit (New England Biolabs, USA) and concentration was quantified by NanoDrop (Thermo Scientific, USA).

### Knockout construct design and synthesis

Putative *S. alvi* genes identified for knockout were first determined to be non-essential *in vitro* by cross-referencing to the previously published *S. alvi* Tn-seq data set (4). *B. apis* genes were predicted to be non-essential by referencing a previously published Tn-seq study in *Bartonella henselae* (66). Homology arms of 500 or 1,000 bp either flanking or within the gene of interest were determined using the NCBI Gene database, with wkB2 (*S. alvi*) or PEB0150 (*B. apis*) as a reference genome. Knockout constructs were then designed in Benchling’s molecular cloning tool (84), by placing homology arms 5′ and 3′ to an antibiotic resistance cassette, either *kanR*, *specR*, or *ampR*, flanked by a promoter and terminator.

*S. alvi* gene deletion constructs were commercially synthesized either as gBlocks (IDT, USA) or as plasmid inserts (Azenta, USA). *B. apis* gene deletion constructs were made using NEBuilder HiFi DNA Assembly (New England Biolabs, USA). Briefly, DNA 1,000 bp upstream and downstream of *ilvD* was amplified with NEBuilder-designed overhangs, using PEB0150 genomic DNA as a template. Antibiotic resistance cassettes were also amplified (*kanR* was amplified from gfPL152; *specR* was amplified from gfPL151; ampR was amplified from pBTK501 [41]) with NEBuilder-designed overhangs. For each construct, the homology arms and insertion cassette were then assembled as linear dsDNA via NEBuilder HiFi DNA Assembly.

### Fluorescent protein-coding gene insertion construct design and synthesis

Insertion sites in the *S. alvi* genome were identified by finding two regions of the genome deemed non-essential by the *S. alvi* Tn-seq experiment (4). The first was a 378-base-pair intergenic region between the genes *SALWKB2_RS11215* and *SALWKB2_RS11220*. The second was the non-essential gene *clpA*.

Golden Gate assembly part plasmids were made by cloning DNA parts into the entry vector pBTK1001 using BsmBI-v2 (New England Biolabs, USA) (Fig. S5 and S6). Flanking homology sequences of 1,000 bp were PCR amplified from wkB2 genomic DNA with primers that added overhangs, so that they could be cloned into pBTK1001 as Type 1 and Type 5 parts by BsmBI assembly, similar to previously described methods (41). Integration cassettes were constructed as Type 2-3-4 parts in pBTK1001. Two versions of the kanamycin resistance cassette were amplified as Type 2 and Type 4 parts from pBTK1047. The spectinomycin resistance cassette was amplified as a Type 4 part from pBTK520. The *e2-crimson* cassette was amplified as a Type 3-4 part from pLM70. The GFP cassette was amplified as Type 2-3 part from pBTK503. Assembly reactions were heat-shock transformed into NEB 5-alpha *E. coli* cells (New England Biolabs, USA) and plated on selective media. Transformants were cultured overnight. Part plasmids were extracted using a QIAprep Spin Miniprep Kit (Qiagen, Germany) and their sequences were verified by whole plasmid sequencing (Plasmidsaurus, USA). To create plasmids containing an integration cassette flanked by homology to the wkB2 or wkB12 genomes, two homology parts and an integration cassette part were combined with the ColE1 origin-containing pYTK095 backbone (85) using BsaI-HF v2 Golden Gate assembly (New England Biolabs, USA). As before, plasmids were transformed into *E. coli*, transformants were selected, and plasmids were sequence verified.

The fluorescent protein gene insert for *B. apis* was assembled via NEBuilder HiFi DNA Assembly, as described above, using pBTK501 as the template for initial amplification of *gfp*.

### PCR amplification of knockout constructs

Synthesized DNA constructs were amplified via PCR. Briefly, primers with homology to the 5′ and 3′ ends of the construct were designed. For each construct, eight to nine 50 µL PCR reactions were run using these oligos and Q5 or Phusion polymerase (New England Biolabs, USA) for 35–36 cycles. A sample was run on a 1% agarose gel and imaged via UV transilluminator, to confirm successful amplification. Amplicons were purified using Magbeads (Axygen, USA) or a DNA Clean & Concentrator kit (Zymo Research, USA), and concentration was measured via NanoDrop (Thermo Scientific, USA). Under these conditions (9 × 50 µL PCRs), 20–30 μg of DNA was obtained, enough for performing multiple 5 µg electroporations if desired.

### *Snodgrassella* electroporation

Electrocompetent *S. alvi* cells were prepared prior to transformation in a manner similar to that commonly used for *E. coli*. Briefly, *S. alvi* was grown on solid media, as described above. After the appearance of colonies, cells were scraped and resuspended in a cell culture flask containing 50 mL Columbia broth. Cells were incubated without shaking at 35°C, 5% CO_2_ for 2–3 days. Working at room temperature, cells were then pelleted at approximately 5,000 × *g* for 10 min and supernatant decanted. Cells were washed two times by resuspending in 30 mL Ultrapure dH_2_O and pelleting, as above. After the third centrifugation, cells were resuspended in 1 mL filtered 10% glycerol in dH_2_O, dispensed into 100 µL aliquots, and stored at −80°C.

*S. alvi* cells were electroporated in a manner similar to that used for *E. coli*, with a few modifications. Briefly, electrocompetent cell preps were first thawed on ice. (Note, freezing electrocompetent cells prior to electroporation was done for convenience and is not required for successful electroporation.) Electrocompetent cells were incubated with approximately 500 ng of plasmid DNA or 5–10 μg of knockout/insertion construct DNA for 20 min (5 µg of DNA was used to construct most knockout strains; 10 µg of DNA was used to construct PL269 and PL271). The cell + construct mixture was added to either a 1 or 2 mm electroporation cuvette depending on the volume. Cells were electroporated with a Gene Pulser Xcell Electroporator (Bio-Rad, USA) using 1.8 kV (for 1 mm cuvettes) or 3 kV (for 2 mm cuvettes). Cells were resuspended in Columbia broth and allowed to recover without antibiotics overnight at 35°C, 5% CO_2_. The next day, cells were pelleted, resuspended in 100 µL of media, and the entire volume was plated on Col-B agar plates with the respective antibiotic. Plates were incubated for 2–3 days at 35°C, 5% CO_2,_ until the appearance of colonies. Multiple colonies were then isolated by passage onto fresh Col-B agar plates, which were allowed to form colonies.

*S. communis* cells were grown on solid media as described above. After the appearance of colonies, cells were scraped and resuspended in 1 mL of sterile ultrapure water at room temperature. Cells were pelleted by centrifugation at 5,000 × *g* for 10 min and supernatant was decanted. Cells were washed one more time in sterile ultrapure water and pelleted as above. Cells were finally resuspended in 1 mL filtered 10% glycerol in dH_2_O, dispensed into 100 µL aliquots, and stored at −80°C. Cells were electroporated as described for *S. alvi* using 500 ng of plasmid. Cells were resuspended in Columbia broth and allowed to recover without antibiotic overnight at 35°C, 5% CO_2_. The next day, cells were pelleted, resuspended in 100 µL of media, and the entire volume was plated on Col-B agar plates with the respective antibiotic. Plates were incubated for 4–5 days at 35°C, 5% CO_2,_ until the appearance of colonies. Multiple colonies were then isolated by passage onto fresh Col-B agar plates, which were allowed to form new colonies.

For preparation of *B. apis* electrocompetent cells, *B. apis* grown on solid media was scraped and resuspended in 1 mL filtered 10% glycerol in dH_2_O. Cells were pelleted two times as above, resuspended in 10% glycerol in dH_2_O, and dispensed into 60 µL aliquots. Cells were electroporated as described above using 5 µg of DNA or no DNA. Electroporants were recovered in Insectagro DS2 (Corning, USA) overnight at 35°C, 5% CO_2_, plated on selective Col-B agar, and incubated at 35°C, 5% CO_2_ for 3–4 days. Transformants were isolated prior to creation of glycerol stocks.

### Recombination efficiency and frequency determination

Across all the constructed strains described here, PCR of transformants confirmed insertion in all but four clones (the PCR reaction failed in these four clones). In *ilvD::gfp-ampR*, recombination accuracy by comparison of fluorescent to non-fluorescent colonies was 99.5%. Therefore, for recombination efficiency and frequency calculations, all transformants were simply counted as recombinants.

Recombination efficiency was determined by counting the number of transformants on selection plates, multiplying by any dilution factor, then dividing by μg of DNA used for transformation. To calculate recombination frequency, a fraction of the electroporation volume was plated in a 10-fold dilution series on plain media. CFU were counted and after multiplying by the dilution factor, total cells in the electroporation reaction were determined. Recombination frequency was then calculated by dividing transformant CFU by total cells in a given reaction.

### PCR screen for knockout mutants

Colony PCR was performed to screen for proper genomic insertion of the knockout/insertion constructs. Briefly, 2–14 colonies were picked from the transformation isolation plates and resuspended in 20 µL of dH_2_O. PCR was run using diluted template, oligos flanking the antibiotic resistance cassette, and Q5 polymerase. A control PCR was also run using WT wkB2 or PEB0150, depending on the experiment. A portion of the PCR reaction was run on a 1% agarose gel and imaged using a UV transilluminator to visualize DNA band size. Amplicons of interest were prepared using magnetic beads (Axygen, USA) and concentration was quantified via NanoDrop (Thermo Scientific, USA). Amplicons were sent for Sanger or Nanopore amplicon sequencing. Sequences were aligned to the designed knockout construct reference using Benchling’s molecular cloning tool to confirm the presence of the antibiotic resistance cassette.

### Knockout/insertion confirmation by WGS

We sequenced strains with genome modifications using either short-read (Illumina) or long-read (Oxford Nanopore) technologies. For short-read sequencing, genomic DNA (gDNA) was purified from the relevant strain using a DNeasy Blood & Tissue kit (Qiagen, Germany). For long-read sequencing, high molecular weight gDNA was isolated using the Quick-DNA HMW MagBead kit (Zymo Research, USA). gDNA concentrations were measured by Qubit (Invitrogen, USA) or NanoDrop (Thermo Scientific, USA).

gDNA was then sequenced in one of three ways, depending on availability of in-house resources: (i) commercial library preparation and short-read sequencing (SeqCenter, USA); (ii) in-house long-read sequencing; or (iii) in-house library preparation and commercial short-read sequencing (Psomagen, USA). Thus, each strain was sequenced using either Illumina or Oxford Nanopore, not both. Commercial library preparation used the tagmentation DNA Prep Kit (Illumina, USA). For in-house short-read sequencing library preparation, 10 ng gDNA was input into the xGen DNA lib Prep EZ kit using the xGen Deceleration module (Integrated DNA Technologies, USA). All reactions were carried out at 20% of the manufacturer’s recommended volumes, with dual 6-bp indexes incorporated during a final 12-cycle PCR step. For long-read sequencing, 175 ng of gDNA was prepared using a Rapid Barcoding Sequencing Kit and sequenced on an R9.4.1 flow cell using a MinION MK1C instrument (Oxford Nanopore, UK). Basecalling was done using Guppy (v6.3.9) in fast mode. Long-read FASTQ files were trimmed using Porechop (v0.2.4) (86) with the option to discard reads with internal adaptors. Short-read FASTQ files were trimmed using fastp (v.0.23.4) (87).

Presence of each desired mutation was determined using breseq (v0.38.1) (88). breseq works by aligning reads obtained from WGS (Illumina or Nanopore) to a reference genome. breseq outputs a variant calling report, which highlights differences between the aligned runs and the reference genome, including mutations, missing coverage, and new junctions. For engineered strains, we used both the chromosome and antibiotic resistance cassette or plasmid as templates for variant calling with breseq. The output includes missing coverage from the genome and new junctions between the antibiotic cassette and genome. These two pieces of information allow for verification both that a gene of interest was deleted and that the antibiotic resistance cassette was inserted into the correct location. Accordingly, variant calling was performed on trimmed FASTQ files using breseq with the *S. alvi* wkB2 genome (GenBank: CP007446) (89)*, S. alvi* wkB9 genome (GenBank: CP132375) (90), *S. alvi* wkB332 genome (GenBank: MEIJ01000000) (91), *S. communis* wkB12 genome (GenBank: JFZW01000000) (89), or the *B. apis* PEB0150 genome (GenBank: LXYS01000000) (92), and the appropriate replacement cassette or plasmid sequences as references. We sequenced multiple strains constructed from the same *S. alvi* wkB2 and *B. apis* PEB150 stocks, which let us differentiate secondary mutations that occurred during genome editing from discrepancies that could be mutations shared by our stocks or errors in the reference sequences for these strains. Because Nanopore sequencing has a limited ability to correctly determine the number of bases in longer homopolymer stretches, putative indels in homopolymers of 3+ bases were not reported for the analysis of Nanopore data.

### Crystal violet biofilm formation assay

Biofilm formation in *S. alvi* was assayed using a crystal violet assay, as previously described (4, 50). Briefly, *S. alvi* strains were inoculated directly from a freezer stock into an overnight culture in a 50 mL conical containing Insectagro DS2 + antibiotic, and incubated for 3 days at 35°C, 5% CO_2_. 1:40 dilutions of confluent cells (including resuspended biofilm) were made in Insectagro DS2 without antibiotics in a 96-well plate. (Note that outer wells were filled with either blank media or culture that would not be used in downstream analysis, due to differential growth resulting from differential oxygen availability.) Plates were incubated for 2 days at 35°C, 5% CO_2_. After 2 days, OD_600_ was measured on a Spark 10M plate reader (Tecan, Switzerland) to quantify overall cell growth. Plates were washed three times by dunking in dH_2_O and stained in 0.1% crystal violet (in dH_2_O) for 10 min. Stain was then removed and plates were allowed to dry in a warm room (37°C) for 1–3 h. Dried plates were then imaged. 30% acetic acid (in dH_2_O) was added to resolubilize the dried crystal violet and incubated for 10 min. OD_550_ was then measured on a Spark 10M to quantify the amount of biofilm present.

Plate reading data were analyzed by first subtracting background (blank reading) in the OD_600_ and OD_550_ readings. Outer wells were omitted from the analysis (see above) and OD_600_ values of less than 0.01 were removed. OD_550_ was then divided by OD_600_, and OD_550_/OD_600_ was plotted in Prism Graphpad (Dotmatics, USA) for each strain. Only positive OD_550_/OD_600_ values are displayed. A Shapiro-Wilk Normality test was run to determine if each sample was normally distributed, and each condition passed. A one-way parametric ANOVA with Dunnett’s multiple comparison test (comparing to the WT control) was subsequently performed, and significance was indicated on plots.

### Imaging and quantification of fluorescent strains

Fluorescent protein gene insertion strains were imaged and/or quantified for fluorescence. wkB2 + genomic *gfp* and wkB2 were grown for 2 days on Col-B agar + kanamycin media at 35°C, 5% CO_2_. After 2 days, cultures were scraped, resuspended in PBS in microcentrifuge tubes, and imaged in front of a blue light transilluminator (470 nm). For experiments involving E2-Crimson, wkB2 + genomic *e2-crimson*, wkB2 + pSL1, and wkB2, cultures were grown for 2 days in liquid BHI media in a sealed bag maintaining a microaerobic environment (85% nitrogen; 10% CO_2_; 5% O_2_) (93). After 2 days, cultures were transferred to a 96-well plate and read in a Tecan Infinite 200 PRO plate reader in triplicate, using 611 nm excitation and 646 nm emission (94) with optimal gain determined by the plate reader. Cultures were then pelleted, resuspended in PBS, and transferred to a 96-well plate. Cultures in PBS were similarly read in the plate reader using 611 nm excitation and 646 nm emission. Absorbance readings from technical replicates were averaged, then log_10_-transformed and plotted in GraphPad Prism , with individual data points for each group representing biological replicates. A Shapiro-Wilk Normality test was run to determine if each sample was normally distributed, and each condition passed. A one-way parametric ANOVA with Tukey’s multiple comparison test was subsequently performed and significance was indicated on plots.

For imaging of wkB12 + *gfp* cells, wkB12 and wkB12 *gfp-specR* strains were grown for 3 days on Col-B agar + spectinomycin media at 35°C, 5% CO_2_. Cells were scraped, resuspended in PBS in microcentrifuge tubes, and imaged in front of a blue light transilluminator (470 nm). For fluorescence quantification, cells resuspended in PBS were diluted to an OD_600_ of 1.0 and transferred to a 96-well plate. Fluorescence intensity was read in a Tecan M200 Infinite plate reader in triplicate, using 485 nm excitation, 535 nm emission and manual gain set at 90. Absorbances at 600 nm (OD_600_) were also measured. Fluorescence reads were normalized for OD_600_. These data were log_10_-transformed and tested for normality with a Shapiro-Wilk Normality test. An unpaired parametric *t* test was performed and significance was indicated on the plot.

Fluorescence of *B. apis ilvD::gfp-ampR* on transformation plates was visualized by imaging plates in front of a blue light transilluminator (470 nm).

### Cell growth rate and *in vitro* viability determination

Growth rate was determined by measuring the change in OD_600_ over time for strains of interest. Briefly, six independent 3-day-old overnight cultures per condition were OD_600_-normalized in Insectagro DS2. Cultures were grown in technical triplicate in a 96-well plate in a Spark 10M at 35°C, 5% CO_2_. OD_600_ values were taken at 1-h intervals for 72 h. Grow curve data were analyzed by plotting the means of independent cultures as individual curves (the same color was used for replicates from the same condition). Technical replicates were plotted as vertical lines.

Cell viability was determined by measuring CFU/mL for strains for interest. Six independent 3-day-old overnight cultures per condition were OD_600_-normalized in Insectagro DS2. Cultures were then diluted in a 10-fold dilution series, spotted on Col-B agar, and incubated at 35°C, 5% CO_2_ for 2–3 days. After the appearance of colonies, CFU were counted and CFU/mL were calculated. CFU/mL data were log_10_-transformed, then tested for normality by a Shapiro-Wilk test. An unpaired parametric *t* test was performed on the log_10_-transformed data to assess the significance of any differences between group means.

### Honey bee gut colonization and *in vivo* viability determination

Honey bees were colonized with wkB2 or engineered *S. alvi* as previously described (28). Briefly, a caged frame with capped pupae was brought to the lab and incubated overnight at 35°C and 80% relative humidity. The next day, newly emerged adults (NEWs) were collected. Col-B plates containing wkB2 or engineered *S. alvi* were grown at 35°C, 5% CO_2_ for 2–3 days. On the day of colonization, cells were scraped from these plates, resuspended in PBS, and added to a filter-sterilized 1:1 sucrose:water solution. NEWs were coated with and fed the bacteria/feed solution to inoculate them with *S. alvi*. Inoculated NEWs were incubated in cup cages at 35°C and 80% relative humidity with pollen and sucrose solution for 7 days. On day 7, live bees were immobilized by chilling at 4°C for 30 min. Guts were pulled from bees on ice and the ilea were dissected. Ilea were homogenized in PBS with a pestle, diluted in a 10-fold series, then plated on Col-B + tetracycline or Col-B + tetracycline + kanamycin agar plates. Plates were grown at 35°C, 5% CO_2_ for 2–3 days, then colonies counted and CFU/ileum calculated. CFU/ileum data were log_10_-transformed, then tested for normality by a Shapiro-Wilk test. A Mann-Whitney *U* test was performed on the log_10_-transformed data to assess the significance of any differences between group medians.

## Data Availability

Raw FASTQ files are available from the NCBI Sequence Read Archive (PRJNA1017617).

## AFTER EPUB

*[This article was published on 6 August 2024 with an incorrect supplemental file. The supplemental material was corrected in the current version, posted on 11 September 2024.]*

## SUPPLEMENTAL MATERIAL

Supplemental material mbio.01392-24-s0001.pdf Figures S1 to S16.

Table S1 mbio.01392-24-s0002.xlsx Transformant and recombinant quantification.

Table S2 mbio.01392-24-s0003.xlsx WGS variant calling.

Table S3 mbio.01392-24-s0004.xlsx Secondary mutations in engineered strains.

Table S4 mbio.01392-24-s0005.xlsx Strains, plasmids, gene fragments, and oligonucleotides.

ASM does not own the copyrights to Supplemental Material that may be linked to, or accessed through, an article. The authors have granted ASM a non-exclusive, world-wide license to publish the Supplemental Material files. Please contact the corresponding author directly for reuse.
