# Supplementary material for: One-step genome engineering in bee gut bacterial symbionts
Source: mBio. 2024 Aug 6;15(9):e01392-24. doi: 10.1128/mbio.01392-24 (PMC11389375; doi:10.1128/mbio.01392-24)
Supplement: Supplemental material — Figures S1 to S16. [file mbio.01392-24-s0001.pdf]

Supplemental material

Table S1. Transformant and recombinant quantification (see excel doc)

Table S2. WGS variant calling (see excel doc)

Table S3. Secondary mutations in engineered strains (see excel doc)

Table S4. Strain, plasmid, gene fragment, oligo lists (see excel doc)

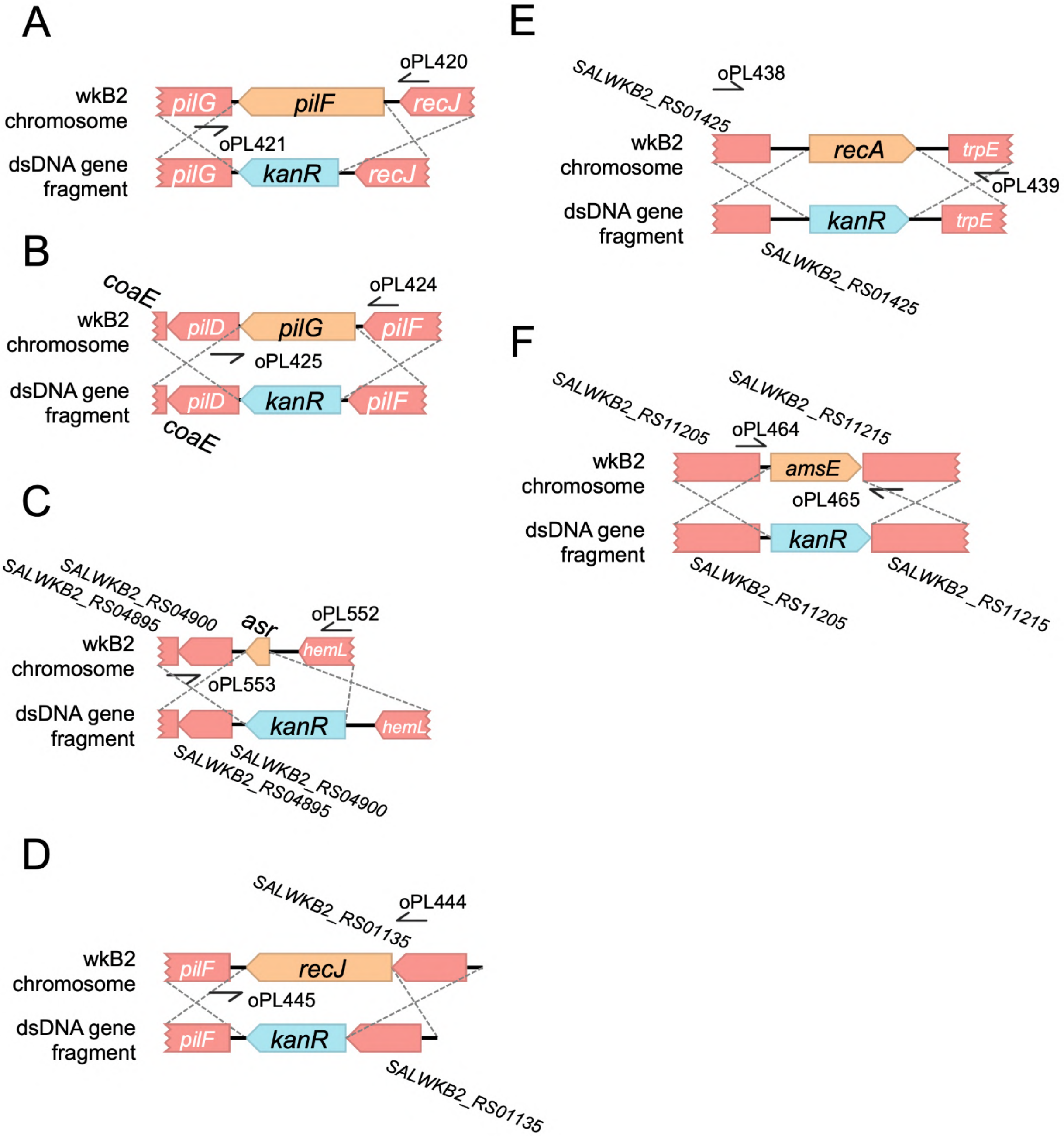

Fig S1. Construct design of additional *S. alvi* gene knockouts

A,B,C,D,E,F. Deletion maps of indicated genes in their genomic contexts: *pilF* (A), *pilG* (B), *asr* (C), *recJ* (D), *recA* (E), *amsE* (F). For each gene, deletion construct and screening primers are depicted.

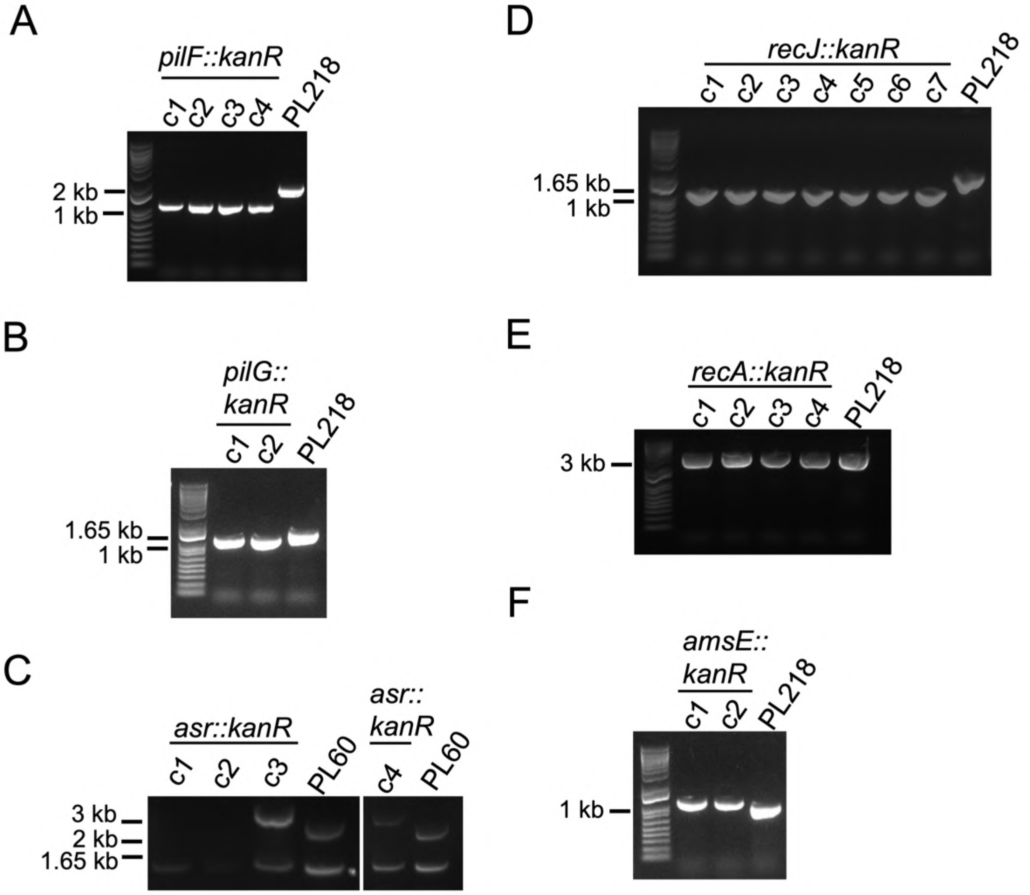

Fig S2. Knockout of nearly all additional genes is accurate

DNA gels of PCR screen of additional knockout strains. PCR products of clones of each knockout and a WT control [PL60 (wkB2) or PL218 (wkB2 + pDS-GFP)] were run with the corresponding primers (shown in Fig S2.)

A. A 1000 bp band corresponding with *kanR* is observed in 4 out of 4 *pilF::kanR* clones, compared to the 2000 bp control band, indicating 100% editing accuracy.

B. A 1000 bp band corresponding with *kanR* is observed in 2 out of 2 *pilG::kanR* clones, compared to the 2000 bp control band, indicating 100% editing accuracy.

C. A ~3000 bp band corresponding with *kanR* + flanking regions is observed in 2 of the 4 tested *pilG::kanR* clones, compared to the 2207 bp control, indicating 50% editing accuracy. Note, a ~1500 bp off-target band is seen in both experimental and control sample PCR reactions, likely arising due to inefficient PCR.

D. A 1000 bp band corresponding with *kanR* is observed in 7 out of 7 *recJ::kanR* clones, compared to the 1755 bp control band, indicating 100% editing accuracy.

E. A ~3000 bp band is observed in *recA::kanR* clones and control sample. As both *kanR* and control bands are predicted to be ~3000 bp, editing accuracy is not evident from PCR alone.

F. A band just over 1000 bp corresponding with *kanR* is observed in 2 out of 2 *amsE::kanR* clones, compared to the 922 bp control band, indicating 100% editing accuracy.

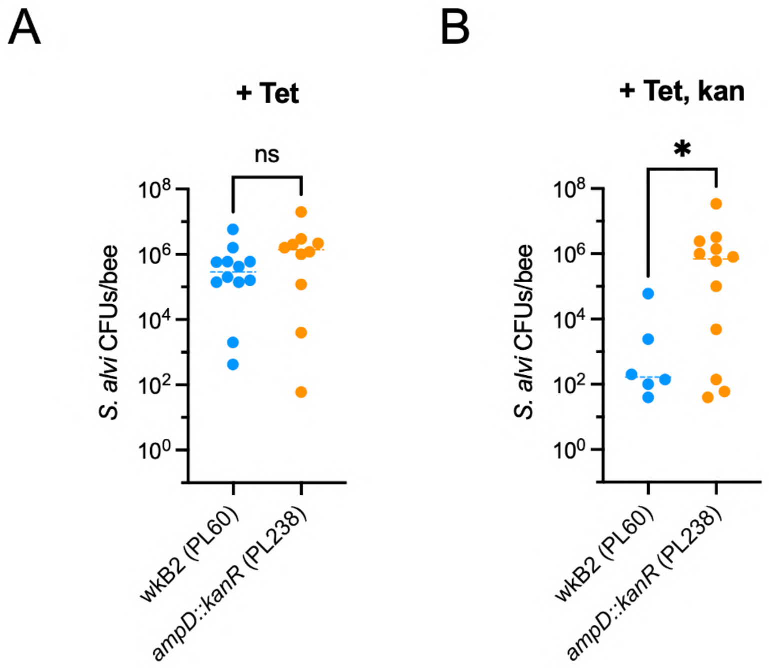

Fig S3. Secondary mutations in *ampD::kanR S. alvi* cells do not affect bee host colonization

A. Plot of CFUs/bee ileum on Col-B +tetracycline plates to quantify colonization of wkB2 and *ampD::kanR S. alvi* cells in honey bees, demonstrating that secondary mutations present in *ampD::kanR* cells do not adversely affect host colonization. Dotted line = median; N=12 for each condition. No significant difference between the log-transformed means of the groups was found by a Mann–Whitney *U* test (P=0.2087).

B. Plot of CFUs/bee ileum on Col-B +tetracycline, kanamycin plates to quantify colonization of wkB2 and *ampD::kanR S. alvi* cells in honey bees, demonstrating that *ampD::kanR* growth is not due to wkB2 contamination. Dotted line = median; N=12 for each condition. A significant difference between the log-transformed means of the groups was found by a Mann–Whitney *U* test (P=0.0500).

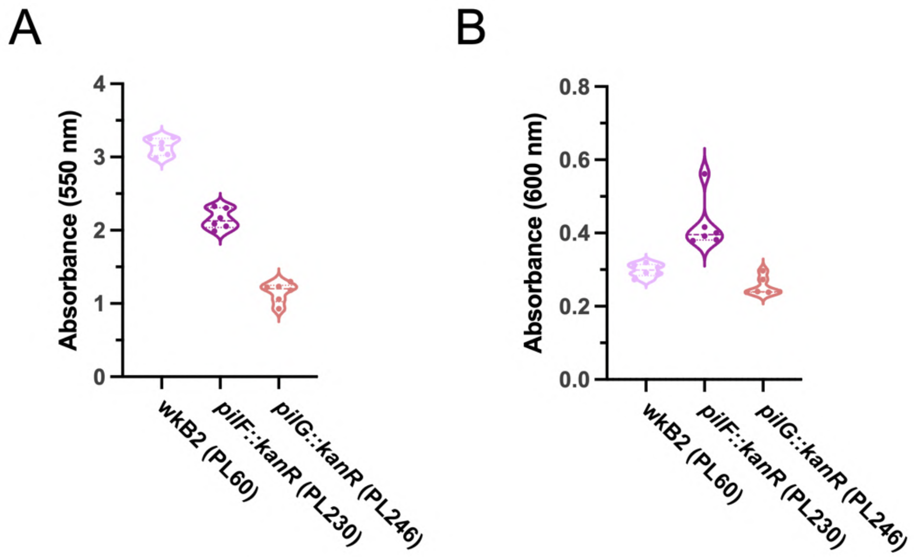

Fig S4. Biofilm formation is impaired in mutants, with limited impact to growth

A. Plot of OD_{550} to quantify biofilm formation for wkB2 and biofilm knockout strains, demonstrating that *pilF::kanR* and *pilG::kanR* have decreased biofilm formation.

B. Plot of OD_{600} to quantify cell growth for wkB2 and biofilm knockout strains, demonstrating minor differences in growth. For A and B, OD_{550} and OD_{600} values were used to calculate OD_{550}/OD_{600} in Fig 3A. Thick dotted line = median; thin dotted line = quartiles. N=6 for each condition.

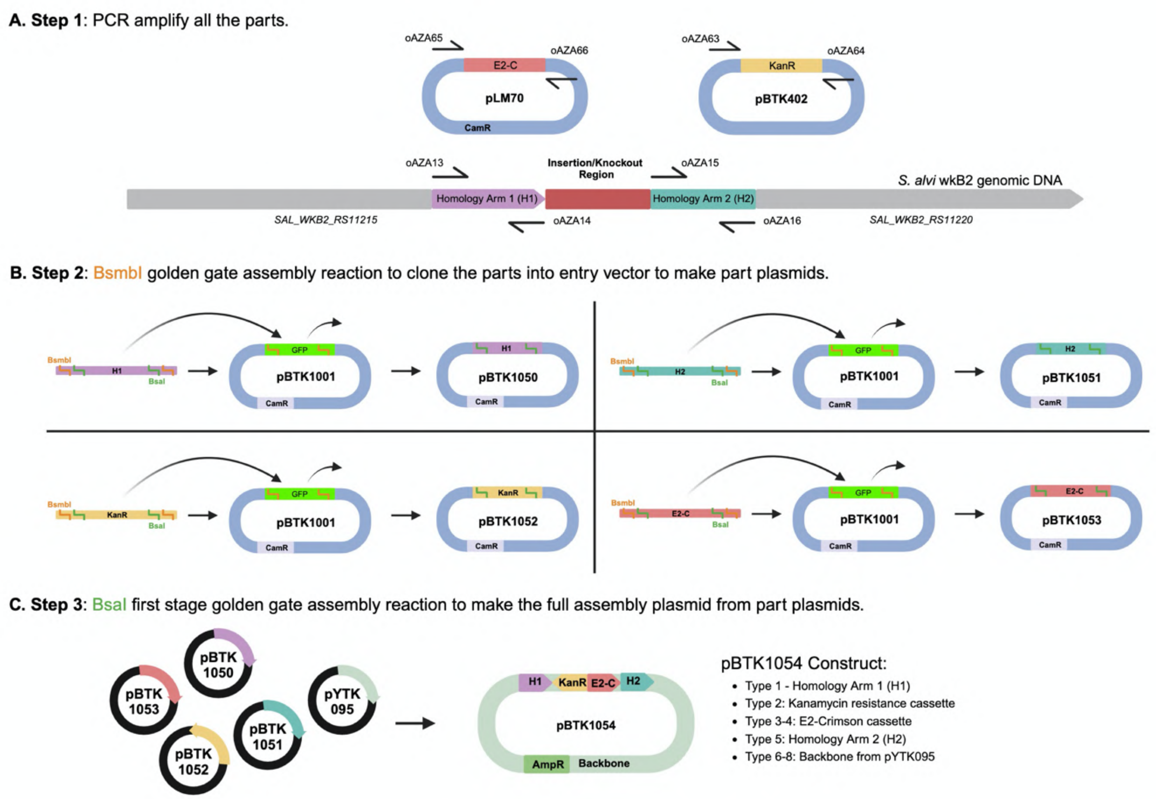

Fig S5. *e2-crimson* genomic insertion plasmid assembly diagram

A. Plasmid and genome maps depicting regions that were used as templates for insert construction. *e2-crimson* (from pLM70), *kanR* (from pBTK402), and homology arms flanking the insertion site (from *S. alvi* genomic DNA) were amplified via PCR. Primers used to amplify these parts contain BsmbI and BsaI recognition and cut sites.

B. Plasmid maps depicting assembly of part plasmids. Parts were cloned into entry vectors (pBTK1001) using BsmbI Golden Gate Assembly mix (NEB, USA) to make part plasmids containing homology arms, *e2-crimson*, and *kanR*.

C. Plasmid maps depicting final assembly of the part plasmids (Type 1-8) using BsaI first-stage Golden Gate Assembly mix (NEB, USA) to create the *e2-crimson* genomic insertion plasmid .

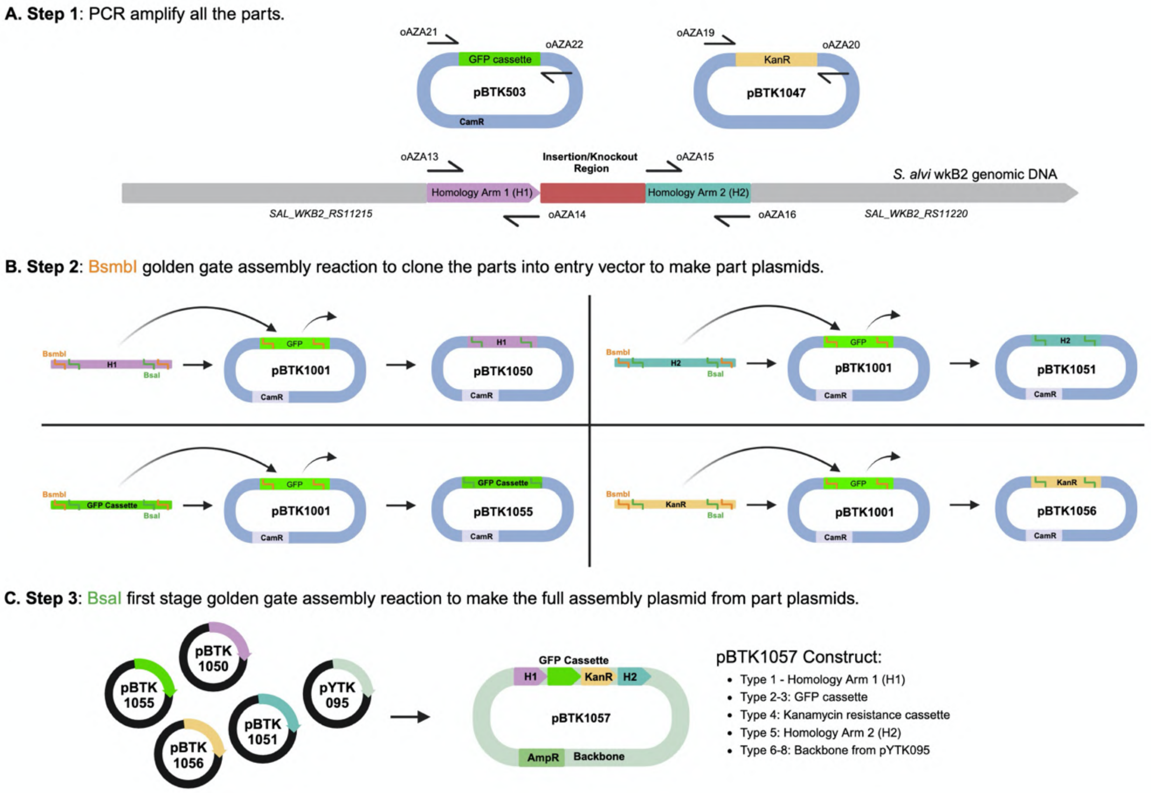

Fig S6. *gfp* genomic insertion plasmid assembly diagram

A. Plasmid and genome maps depicting regions that were used as templates for insert construction. *gfp* (from pBTK503), *kanR* (from pBTK1047), and homology arms flanking the insertion site (from *S. alvi* genomic DNA) were amplified via PCR. Primers used to amplify these parts contain BsmbI and BsaI recognition and cut sites.

B. Plasmid maps depicting assembly of part plasmids. Parts were cloned into entry vectors (pBTK1001) using BsmbI Golden Gate Assembly mix (NEB, USA) to make part plasmids containing homology arms, *gfp*, and *kanR*.

C. Plasmid maps depicting final assembly of the part plasmids (Type 1-8) using BsaI first-stage Golden Gate Assembly mix (NEB, USA) to create the *gfp* genomic insertion plasmid.

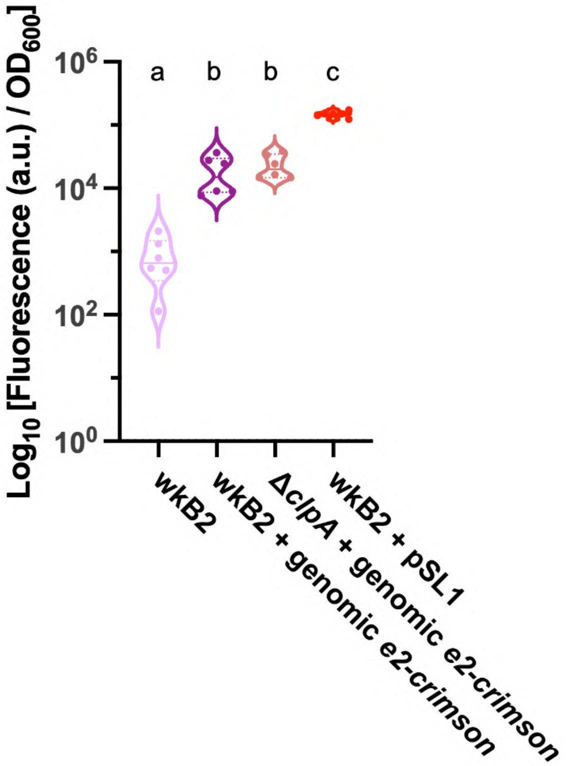

Fig S7. Genomic insertion of *e2-crimson* leads to increase in culture fluorescence
Plot of quantification of *e2-crimson* fluorescence, demonstrating that wkB2 + genomic *e2-crimson* cultures grown in BHI display significantly increased red fluorescence compared to wkB2, and only slightly less fluorescence than wkB2 + pSL1, which contains multiple plasmid copies of *e2-crimson*. Log_{10} of $OD_{611 \text{ ex}, 646 \text{ em}}/OD_{600}$ is shown for cell cultures grown in BHI that were read in a 96 well plate. Solid line = median; thin dotted line = quartiles. N=6 for each condition. Dissimilar letters above each group indicate a significant difference in means ($P < 0.0005$, as determined by One-way parametric ANOVA with Tukey's multiple comparison test).

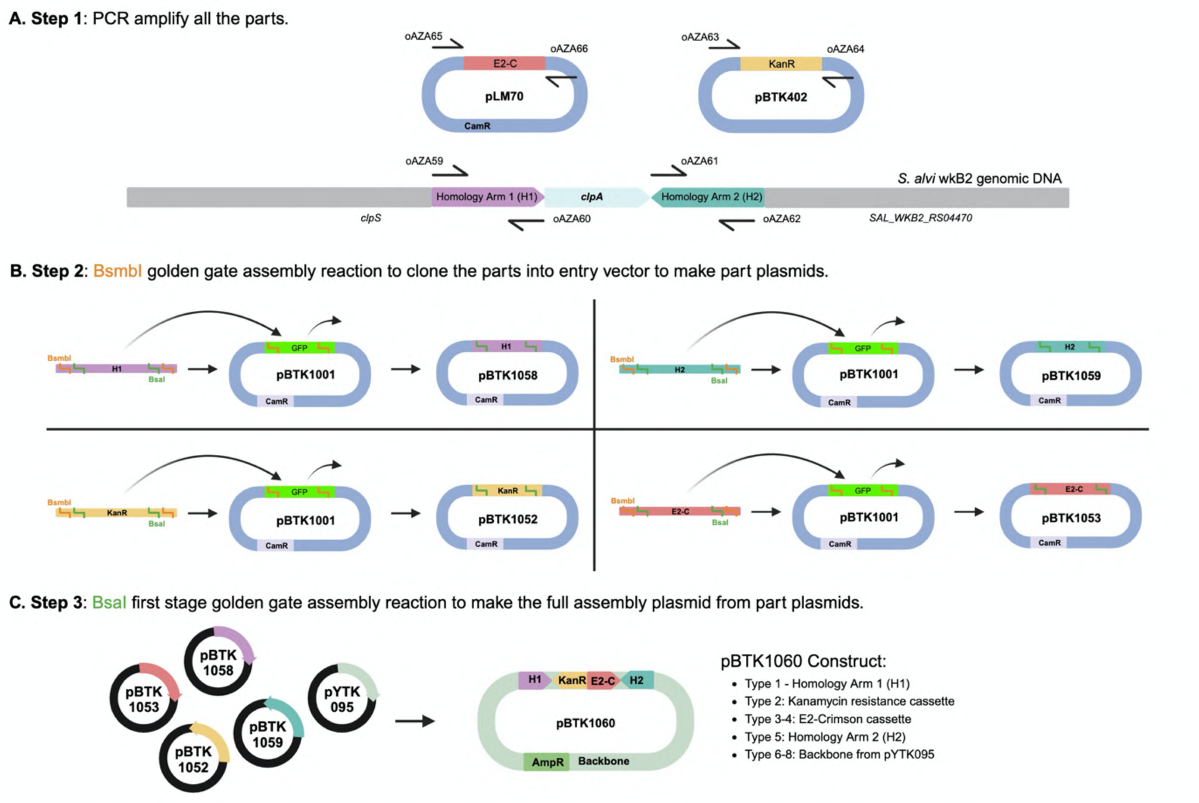

Fig S8. Δ*clpA* + *e2-crimson* genomic insertion plasmid assembly diagram

A. Plasmid and genome maps depicting regions that were used as templates for insert construction. *e2-crimson* (from pML70), *kanR* (from pBTK402), and homology arms flanking *clpA* (from *S. alvi* genomic DNA) were amplified via PCR. Primers used to amplify these parts contain BsmbI and BsaI recognition and cut sites.

B. Plasmid maps depicting assembly of part plasmids. Parts were cloned into entry vectors (pBTK1001) using BsmbI Golden Gate Assembly mix (NEB, USA) to make part plasmids containing homology arms, *e2-crimson*, and *kanR*.

C. Plasmid maps depicting final assembly of the part plasmids (Type 1-8) using BsaI first-stage Golden Gate Assembly mix (NEB, USA) to create the Δ*clpA* + *e2-crimson* genomic insertion plasmid.

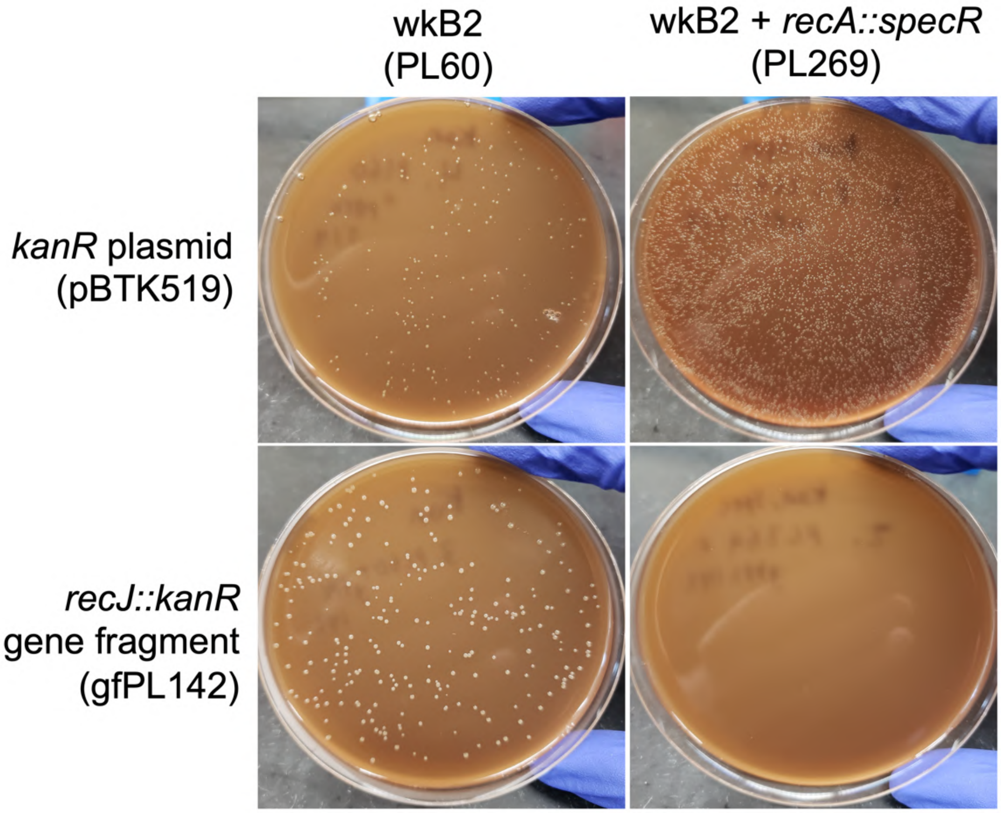

Fig S9. Knockout of additional gene, *recJ*, is not possible in a *recA::specR* background
Images of plates demonstrating that wkB2 (PL60; top left) and wkB2 + *recA::specR* (PL269; top right) are successfully electroporated with a control *kanR* plasmid (pBTK519). The knockout construct *recJ::kanR* (gfPL142) is incorporated into wkB2 (bottom left), but not wkB2 + *recA::specR* (bottom right).

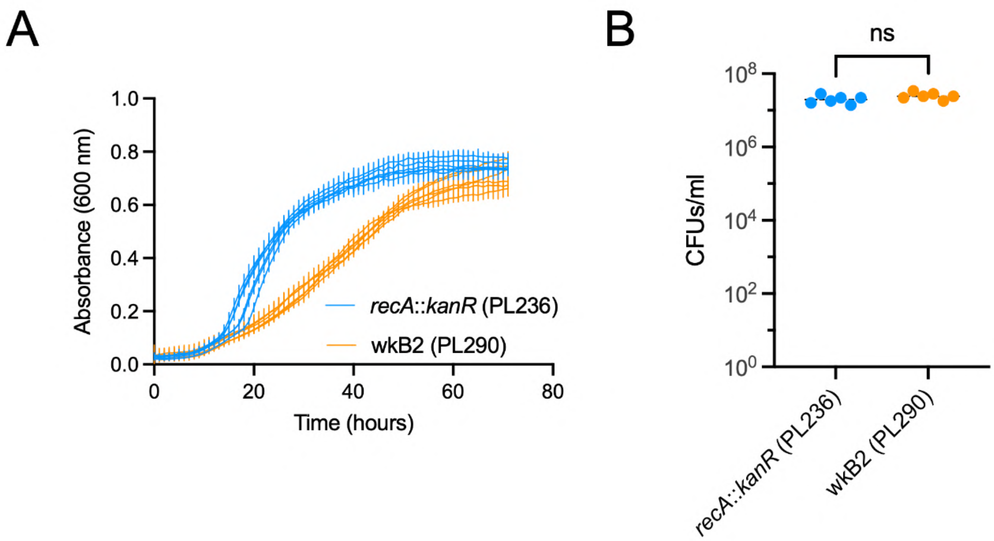

Fig S10. *recA*::*kanR* cells do not have decreased fitness

A. Plot of growth curve, demonstrating *recA*::*kanR* cells have a faster doubling time and slightly higher maximum OD_{600} than wkB2 cells. Six independent cultures were grown for each strain (means plotted as separate curves of the same color). For each independent culture, 3 technical replicates were grown (plotted as vertical lines).

B. Plot of cell viability, demonstrating *recA*::*kanR* cells have similar viability to wkB2 cells when a normalized amount of cells from each are plated on agar. N=6 for each condition. No significant difference between the log-transformed means of the groups was found by an unpaired parametric t test (P=0.1197).

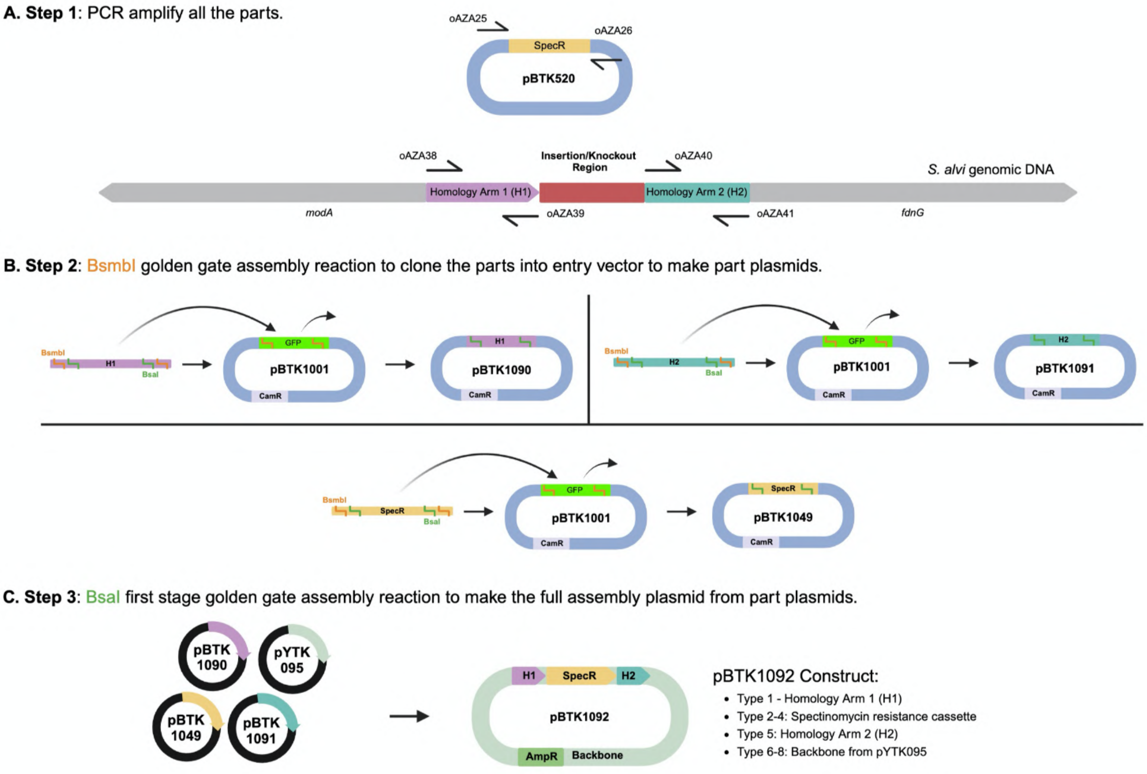

Fig S11. *SpecR* genomic insertion plasmid assembly diagram

A. Plasmid and genome maps depicting regions that were used as templates for insert construction. *SpecR* (from pBTK1049), and homology arms flanking the insertion site (from *S. alvi* genomic DNA) were amplified via PCR. Primers used to amplify these parts contain BsmbI and BsaI recognition and cut sites.

B. Plasmid maps depicting assembly of part plasmids. Parts were cloned into entry vectors (pBTK1001) using BsmbI Golden Gate Assembly mix (NEB, USA) to make part plasmids containing homology arms and *specR*.

C. Plasmid maps depicting final assembly of the part plasmids (Type 1-8) using BsaI first-stage Golden Gate Assembly mix (NEB, USA) to create the *specR* genomic insertion plasmid.

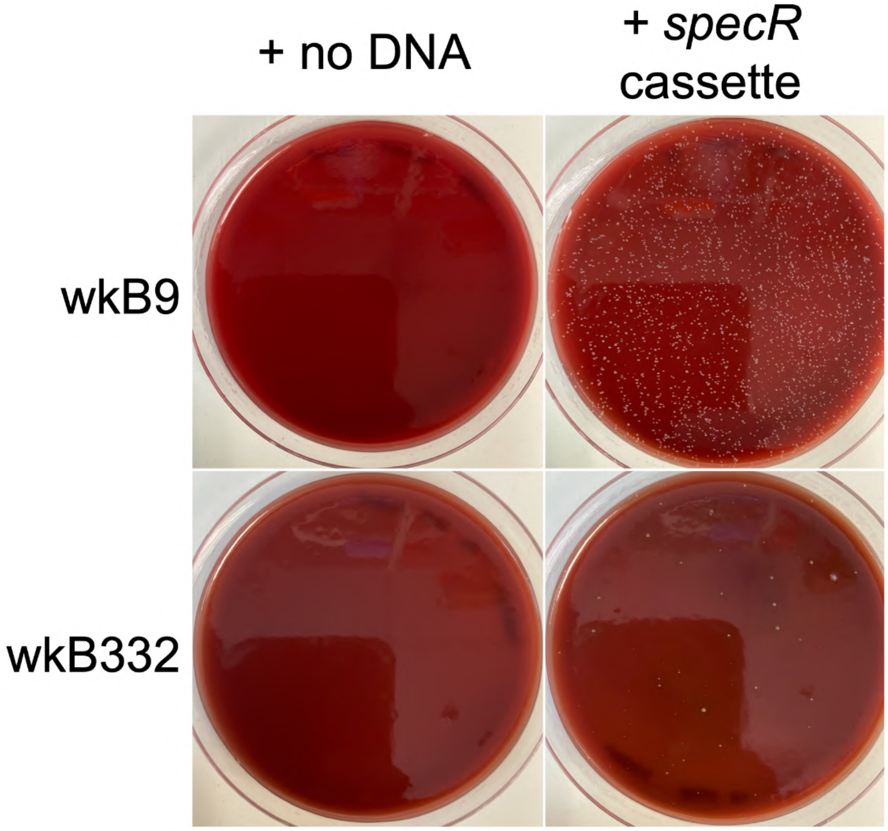

Fig S12. Additional *S. alvi* strains can be engineered with a lightweight appproach
Images of transformation selection plates demonstrating that additional strains of *S. alvi* can be engineered. WT wkB9 (top left) and wkB332 (bottom left) without addition of insertion cassettes do not grow on Col-B + spec agar. Successful transformation of *specR*-containing insertion cassettes into wkB9 (top right) and wkB332 (bottom right) is indicated by colony formation on Col-B +spec agar.

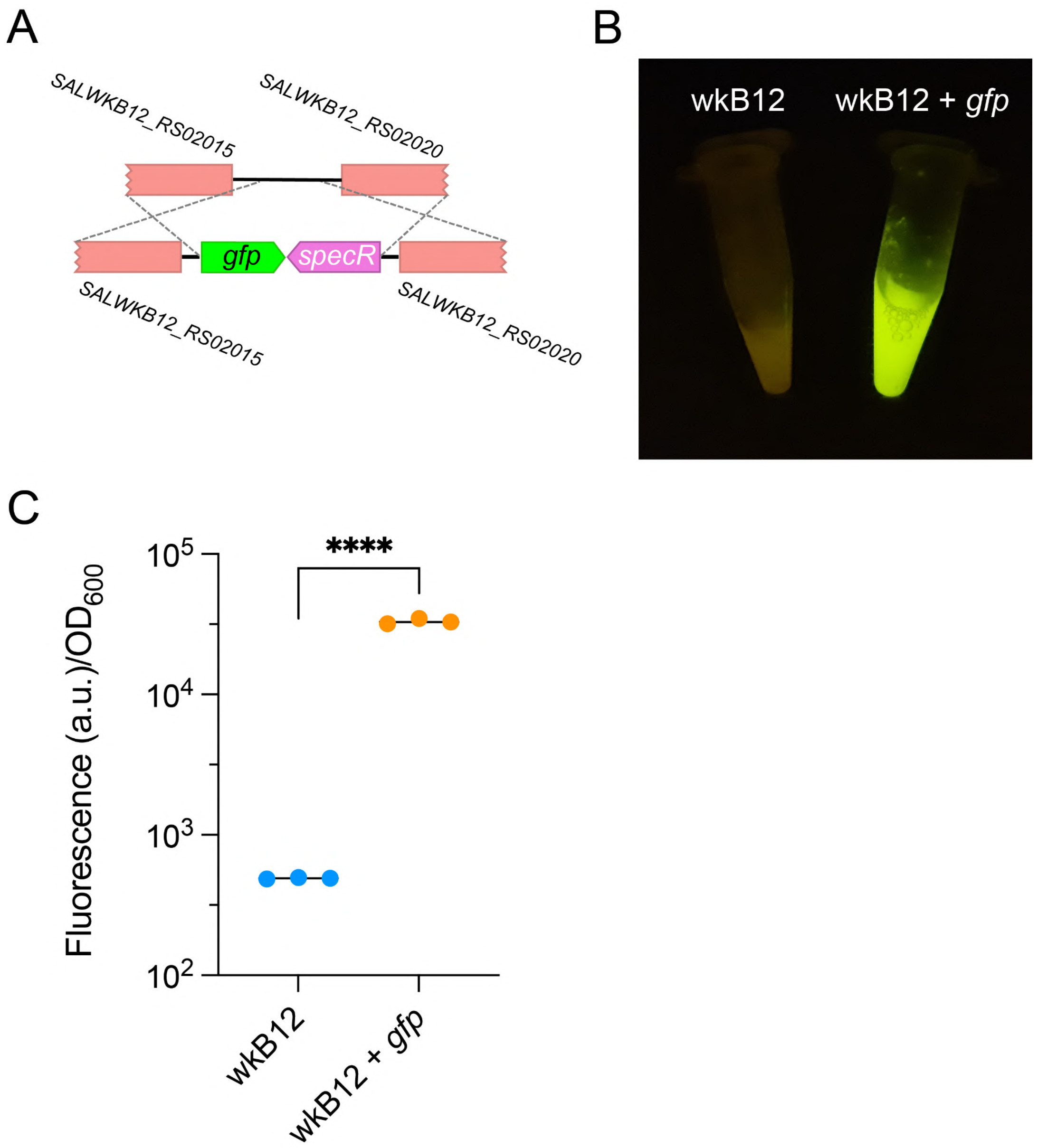

Fig S13. ***S. communis*** **can be engineered with a lightweight approach**

A. Gene insertion map depicting the insertion of a *gfp + specR* cassette in an intergenic region in the *S. communis* wkB12 genome.

B. Image of wkB12 and wkB12 + *gfp* cells in front of a blue light transilluminator (470 nm), demonstrating successful insertion of *gfp*. Green fluorescence is observed in wkB12 + *gfp* cells (right), but not in WT wkB12 cells (left).

C. Plot of quantification of GFP fluorescence, demonstrating that wkB12 + *gfp* cell pellets resuspended in PBS display significantly increased green fluorescence compared to wkB12. Log_{10}-transformed $OD_{485\ ex,\ 535\ em}/OD_{600}$ is shown for cell cultures that were pelleted, resuspended in PBS, and measured for fluorescence ($OD_{485\ ex,\ 535\ em}$) in a 96 well plate reader. Solid line = median; N=3 for each condition. The difference in GFP fluorescence intensity between wkB12 and wkB12 + *gfp* is significant ($P < 0.0001$, as determined by an unpaired parametric t test).

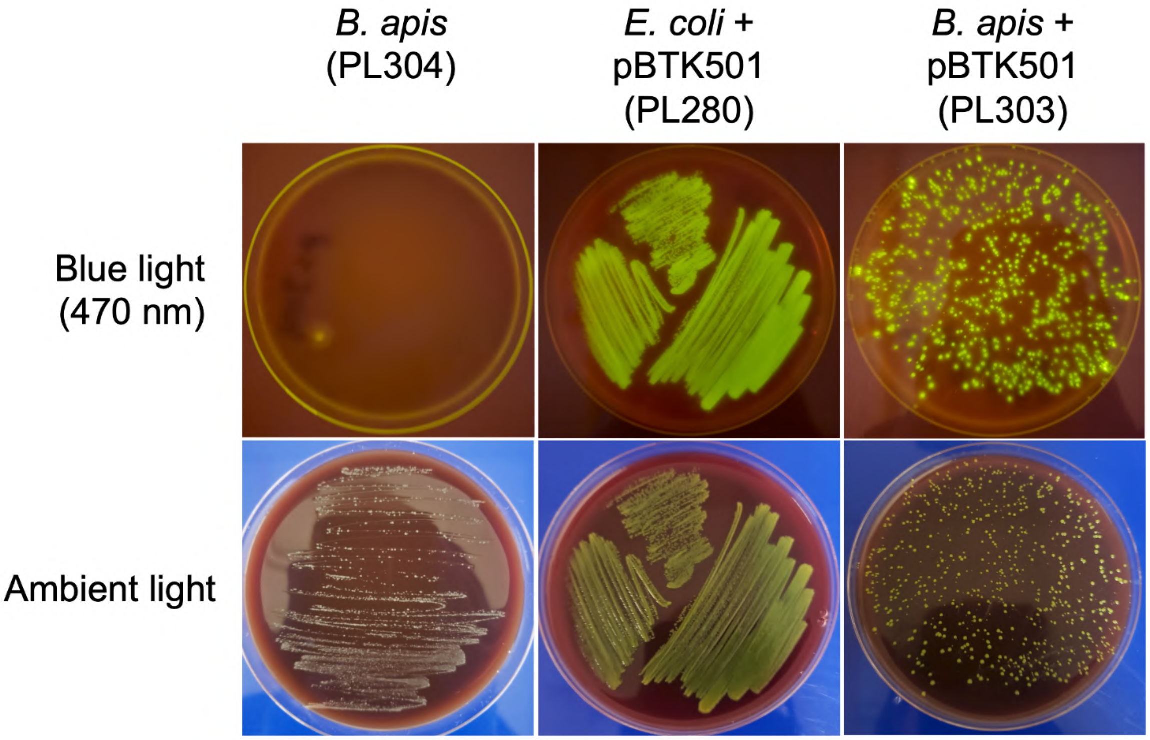

Fig S14. *B. apis* can be transformed with plasmid DNA by electroporation
Images of transformation selection plates demonstrating *B. apis* can be successfully electroporated. WT *B. apis* on plain Col-B agar plates (bottom left) is not fluorescent (top left; single fluorescent spot is plate contamination). *E. coli* containing the *gfp*-expressing plasmid pBTK501 on Col-B + carbenicillin agar plates (bottom middle) is fluorescent (top middle), serving as a positive control for fluorescence. *B. apis* electroporated with pBTK501 and plated on Col-B + carbenicillin agar plates (bottom right) is also fluorescent (top right), indicating successful transformation. All but two colonies are fluorescent.

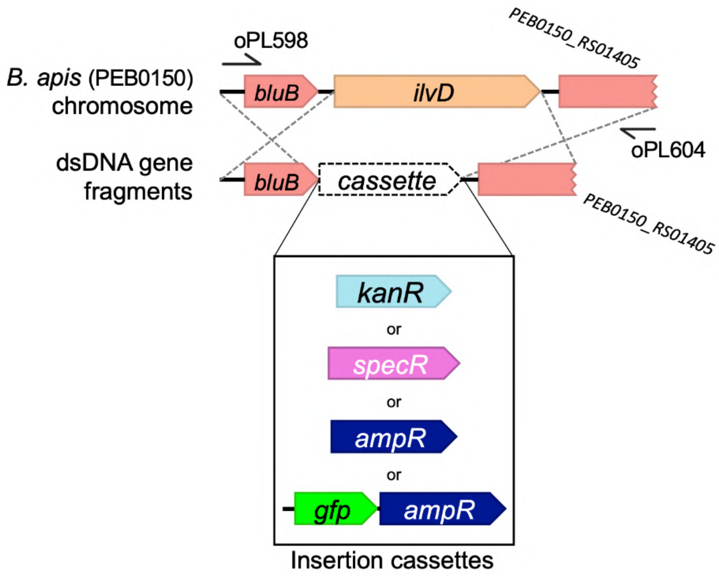

Fig S15. Construct design of *B. apis* gene knockouts

Deletion map of *ilvD* in the *B. apis* genomic context. Four deletion constructs were designed, containing either *kanR*, *specR*, *ampR*, or *gfp-ampR* genes in the region marked "cassette". Screening primers are depicted.

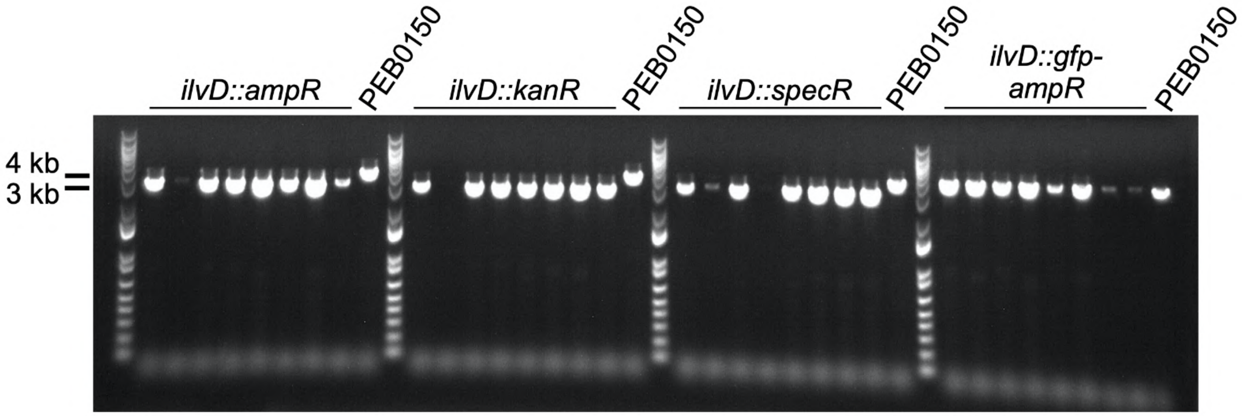

Fig S16. Knockout of *ilvD* in *B. apis* is accurate

DNA gels of PCR screen of *ilvD* knockouts in *B. apis*. PCR products of 8 clones of each knockout and a WT control (PEB0150) were run with the primers oPL598 and oPL604. A 3069 bp band corresponding with *ampR* is observed in 8 out of 8 *ilvD::ampR* clones, compared to the 3970 bp control band (left). A 2974 bp band corresponding with *kanR* is observed in 7 out of 8 *ilvD::kanR* clones, compared to the 3970 bp control band (middle left). A 3128 bp band corresponding with *specR* is observed in 7 out of 8 *ilvD::specR* clones, compared to the 3970 bp control band (middle right). For *ilvD::kanR* and *ilvD::specR*, absence of a band in a single lane suggests a failed PCR reaction. No band shift is expected for *gfp-ampR* compared to *ilvD* control band, so insertion of *gfp-ampR* could be assessed by PCR (right). Insertion of *gfp-ampR* was instead assessed by colony fluorescence (Fig 5).
